# Retro-enantio isomer of angiopep-2 assists nanoprobes across the blood-brain barrier for targeted magnetic resonance/fluorescence imaging of glioblastoma

**DOI:** 10.1038/s41392-021-00724-y

**Published:** 2021-08-19

**Authors:** Ruoxi Xie, Zijun Wu, Fanxin Zeng, Huawei Cai, Dan Wang, Lei Gu, Hongyan Zhu, Su Lui, Gang Guo, Bin Song, Jinxing Li, Min Wu, Qiyong Gong

**Affiliations:** 1 Huaxi MR Research Center (HMRRC), Department of Radiology, Functional and Molecular Imaging Key Laboratory of Sichuan Province, West China Hospital, Sichuan University, Chengdu, China; 2 Research Unit of Psychoradiology, Chinese Academy of Medical Sciences, Chengdu, China; 3 Department of Clinic Medical Center, Dazhou Central Hospital, Dazhou, China; 4 Laboratory of Clinical Nuclear Medicine, Department of Nuclear Medicine, West China Hospital, Sichuan University, Chengdu, China; 5 State Key Laboratory of Biotherapy and Cancer Center, West China Hospital, Sichuan University, and Collaborative Innovation Center for Biotherapy, Chengdu, China; 6 Department of Chemical Engineering, Stanford University, Stanford, California, USA

**Keywords:** Molecular medicine, Drug delivery

## Abstract

Glioblastoma (GBM), one of the most common primary intracranial malignant tumours, is very difficult to be completely excised by surgery due to its irregular shape. Here, we use an MRI/NIR fluorescence dual-modal imaging nanoprobe that includes superparamagnetic iron oxide nanoparticles (SPIONs) modified with indocyanine (Cy7) molecules and peptides (ANG or ^D^ANG) to locate malignant gliomas and guide accurate excision. Both peptides/Cy7-SPIONs probes displayed excellent tumour-homing properties and barrier penetrating abilities in vitro, and both could mediate precise aggregation of the nanoprobes at gliomas sites in in vivo magnetic resonance imaging (MRI) and ex vivo near-infrared (NIR) fluorescence imaging. However, compared with ANG/Cy7-SPIONs probes, ^D^ANG/Cy7-SPIONs probes exhibited better enhanced MR imaging effects. Combining all these features together, this MRI/NIR fluorescence imaging dual-modal nanoprobes modified with retro-enantio isomers of the peptide have the potential to accurately display GBMs preoperatively for precise imaging and intraoperatively for real-time imaging.

## Introduction

Glioblastoma (GBM) is one of the most common malignant brain tumours. At present, surgical operation combined with both radiotherapy and chemotherapy is still the most common treatment method for GBM.^1^ Characterized by heterogeneity and highly invasive growth, the tumour range cannot be accurately located before surgery; thus, the tumours are very difficult to be resected.^2,3^ Unlike the resection of other solid tumours, it is difficult to remove the margins that take up a relatively large space in the brain, while excessive resection may induce damage to the cortical region or the brainstem structure around the tumours, resulting in irreversible neurological injuries.^4^ In addition, the remaining infiltrative glioma cells after surgical resection are nourished by a normal cerebral blood supply, which leads to glioma recurrence.^3^ Therefore, the preoperative identification of the GBM range and rims, accurately outlining the shape of the tumour and precisely locating the lesion area are the fundamental guarantees to achieve accurate tumour resection and minimize nerve function damage.

Magnetic resonance imaging (MRI) is one of the most important preoperative diagnostic methods for intracranial GBMs. To improve the sensitivity of contrast among different tissues, enhanced MRI with contrast agents, such as Gd-DTPA, is used to show a macroscopic region of the GBM.^5,6^ However, using the current enhanced MRI technology, over 10% of GBMs and 30% of undifferentiated astrocytic GBMs cannot be identified. The failure of glioma imaging is mainly attributed to the distinctive characteristics of this intracranial tumour.^7^ The blood-brain barrier (BBB) is an important transit barrier that controls the exchange of substances between the blood and the central nervous system (CNS), maintaining homeostasis of the CNS.^8^ Due to the tight connection between cerebrovascular endothelial cells, which hinders the transport of substances, it is difficult for probes to enter the lesion area in the brain. Similarly, the identification of GBMs with clinical MR contrast agents has limited performance due to their inability to penetrate physiological or pathological BBB. Furthermore, these contrast agents have poor specificity and a short residence time in vivo. Therefore, it is of great significance to develop tumour-targeting imaging probes for the preoperative diagnosis of glioma and accurate location of tumour margins.

With the rapid development of multimodal imaging, the construction of imaging nanoprobes based on molecular biology, nanomaterials and molecular imaging increase the possibility for specific tissue imaging. Multimodal imaging, such as MRI/optical imaging, MRI/radionuclear imaging, and radionuclear imaging/optical imaging, etc., integrates their advantages, overcomes the limitations of a single imaging model and finally improves the clinical imaging specificity and sensitivity.^9–12^ In this study, considering the characteristics of gliomas, we focused on MRI/optical imaging. MRI has a superior temporal and spatial resolution, excellent soft-tissue contrast resolution and no depth limit.^13^ However, its image acquisition time is long, so it is very susceptible to the influence of tissue movement, which produces motion artefacts.^14^ On the other side, optical imaging has the ability of continuous imaging but has poor image resolution and limited light penetration,^14,15^ so its application in intraoperative navigation is relatively limited. Fortunately, near-infrared (NIR) fluorescence imaging, attributed to the properties like reduced light scattering, higher signal-to-noise ratio, relatively higher penetration depth and image resolution,^16^ has been currently popular among the exploitation of intraoperative navigation. Practically, some NIR fluorescent dyes, such as indocyanine green (ICG) and methylene blue, have been approved by Food and Drug Administration for clinic use, indicating great clinical value of NIR fluorescence imaging.^17,18^ In some of the latest studies, a variety of NIR dyes after specific modifications could bring signals to specific lesion areas such as various tumours from different origins,^19,20^ and specific tissues,^16^ to guide the operators for further medical actions. Therefore, by combining these two imaging techniques, MRI and optical imaging, we can obtain both accurate preoperative diagnostic information and intraoperative pathological information to provide guidance for a more precise surgical resection of tumours.

The application of an endothelial cell-mediated endocytosis transport mechanism is an effective strategy for the delivery of probes into the brain. Most small-molecule compounds and polar molecules, such as glucose, amino acids, and short peptides, have specific vectors on endothelial cells.^21–23^ Some particular molecules that cross the BBB can also function as transmembrane transporters by receptor-mediated transfection (RMT) or adsorption-mediated transfection (AMT).^24^ Therefore, the design of cross-BBB shuttle tools should achieve physical material delivery to the brain.

Ideal BBB shuttle carriers exhibit the following characteristics: (1) BBB receptors are highly expressed at the cavity site in cerebrovascular cells; (2) they are able to mediate transcytosis; and (3) they possess high flux and extensive substrate recognition capabilities. Peptides, as ligands, have obvious advantages, such as simple chemical synthesis, low immunogenicity, easy modification, and low influence on the function of delivery.^25^ However, the peptide sequence is commonly linear in structure and is composed of L-amino acids, which makes it easy to be degraded by the protease in vivo and further reduces the targeting efficiency. Fortunately, we can improve the antiprotease degradation function of peptides by utilizing nonnatural amino acids, such as cyclic peptide chains, or the application of D-amino acids.^26–28^ As reported, the retro-enantio isomers of peptides show anti-enzyme degradation properties while preserving the topological characteristics of the sequence, becoming highly enzyme-resistant peptide analogues.^29–31^

The aim of this study is to introduce targeting ligands with high affinity and stability for the construction of imaging nanoprobes. Low-density lipoprotein receptor protein 1 (LRP-1) is highly expressed on brain capillary endothelial cells and glioma cells.^32–34^ Therefore, angiopep-2 (ANG) is a promising ligand to trigger transcytosis.^34^ It not only traverses the BBB but also targets glioma cells by recognizing LRP-1. To enhance the stability of ANG in vivo, we used the retro-enantio isomer of ANG (^D^ANG) to overcome degradation by various enzymes in the blood and cells. Finally, we constructed a new dual-modal imaging probe for effective GBM imaging by combining superparamagnetic iron oxide nanoparticles (SPIONs) with the NIR fluorescent dye indocyanine (Cy 7) and targeting peptides (ANG/^D^ANG).

## Results

### Synthesis and characterization of the peptide/Cy7-SPION probes

**Fig. 1:**
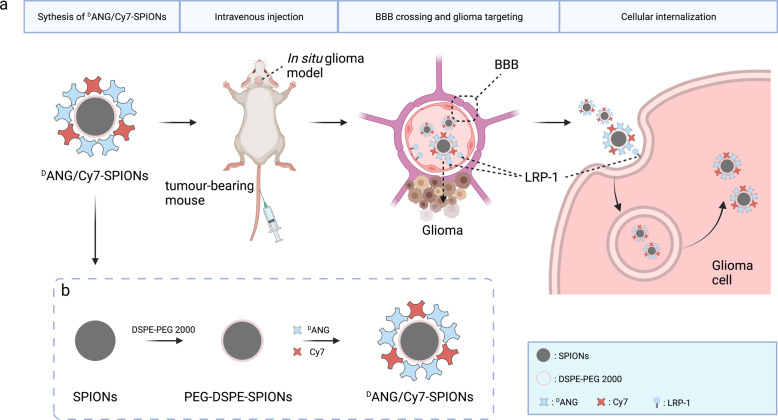
**a** Schematic illustration of the construction and function of ^D^ANG/Cy7-SPIONs, including their mechanism of crossing the BBB and targeting glioma cells and **b** their synthesis process

ANG and ^D^ANG were separated and identified by HPLC and MS. In HPLC analysis, ANG and ^D^ANG peptides reached their single distinctive peaks at 10.344 and 12.088 min, respectively, with their purity reaching 95% (Supplementary Fig. S2b and d). MS analysis revealed that the exact masses of the ANG and ^D^ANG peptides were both 2343.56, which was consistent with their retro-enantio characteristics (ANG: Ac-TFFYGGSRGKRNNFKTEEY-OH, ^D^ANG: Ac-yeetkfnnrkgrsggyfft-OH) (Supplementary Fig. S2a and c). (Fig. 1).

**Fig. 2:**
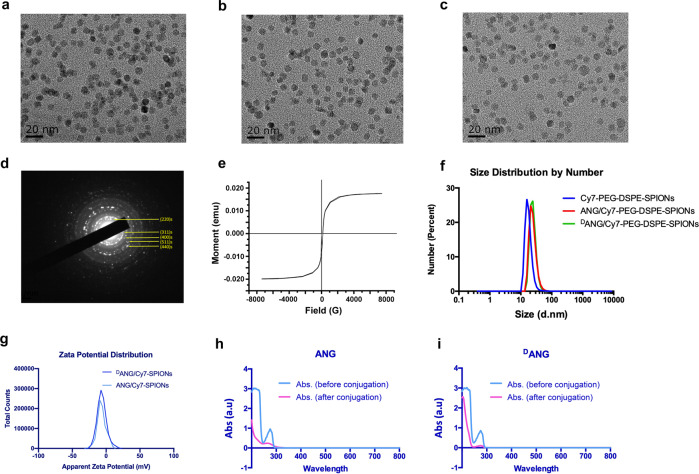
**a** Particle size of Cy7-SPIONs, **b** ANG/Cy7-SPIONs and **c** ^D^ANG/Cy7-SPIONs; **d** Selected area electron diffraction pattern of PEG-DSPE-SPIONs; **e** Magnetic hysteresis curve of NH_2_-PEG-DSPE-SPIONs; **f** Size distribution of Cy7-SPIONs, ANG/Cy7-SPIONs, and ^D^ANG/Cy7-SPIONs; **g** Zeta potential distribution of ANG/Cy7-SPIONs and ^D^ANG/Cy7-SPIONs; **h** UV–vis spectra of ANG and **i** ^D^ANG before and after conjugation

PEG-DSPE-SPIONs were synthesized by the hydrothermal method. Selected area electron diffraction (SAED) imaging showed that the obtained PEG-DSPE-SPIONs had a cubic spinel crystal structure (Fig. 2d), which was similar to polycrystalline-cubic Fe_3_O_4_, consistent with the standard bulk Fe_3_O_4_. The characteristic peaks of iron (Fe), oxygen (O) and carbon (C) were analysed by energy dispersive spectroscopy (EDS) (supplementary Fig. S3c).

Peptide/Cy7-SPIONs probes were successfully produced after the peptides (ANG and ^D^ANG) and Cy7 dyes were conjugated to the PEG terminus through a condensation reaction. UV–vis spectroscopy was used to test whether the peptides had successfully attached to the surface of PEG-DSPE-SPIONs, and the UV–vis absorption spectra of the peptide solutions before and after the reaction were shown in Fig. 2h and i. There was an evident absorption decrease at approximately 280 nm in the spectra of the supernatant solution due to a decrease in amino acids after coupling, which indicated that the PEG-DSPE-SPIONs cores were successfully conjugated with the peptides. Moreover, the coupling efficiency was calculated by measuring the peptide content in the solution collected after the reaction via HPLC, and the results were 8% and 8.7% for ANG and ^D^ANG, respectively. The connection of Cy7 molecules and peptide-PEG-DSPE-SPIONs was detected by a multifunctional fluorescence microplate reader. After connecting the Cy7 molecule, the probes were excited by 720 nm excitation, and there was an obvious emission peak at 820 nm, which indicates successful connection of Cy7 molecules and peptide-PEG-DSPE-SPIONs. For 2 types of peptide-PEG-DSPE-SPIONs (ANG and ^D^ANG) containing 10 mg Fe_3_O_4_, the amounts of Cy7 molecules bound to the probes were 10.03 and 9.87 *µ*g, respectively (supplementary Fig. S3a and b).

After the successful synthesis of the probes, TEM was used to analyse the morphology. All peptide/Cy7-SPION probes had an average size of inorganic core whose diameter was ~10 nm (Fig. 2a–c). In addition, the hydrodynamic size and zeta potential of the probes were measured by dynamic light scattering (DLS) and the results showed that the diameter of the probes after surface modification was slightly larger to various extents because of the presence of DSPE-PEG, peptides and Cy7 molecules (Fig. 2f). Moreover, the probes were electroneutral (Fig. 2g) and their hydrodynamic sizes were 40–50 nm, which was beneficial for their long-term circulation in vivo.

### Magnetic properties and *T*_2_-weighted MR relaxometry

The magnetic hysteresis of PEG-DSPE-SPIONs was measured by VSM at 300 K, which showed that the saturation magnetization (*Ms*) of SPION cores is 78.24 emu/g (Fig. 2e), indicating that the probes exhibited excellent superparamagnetic behaviour and were suitable for further application in MRI.

**Fig. 3:**
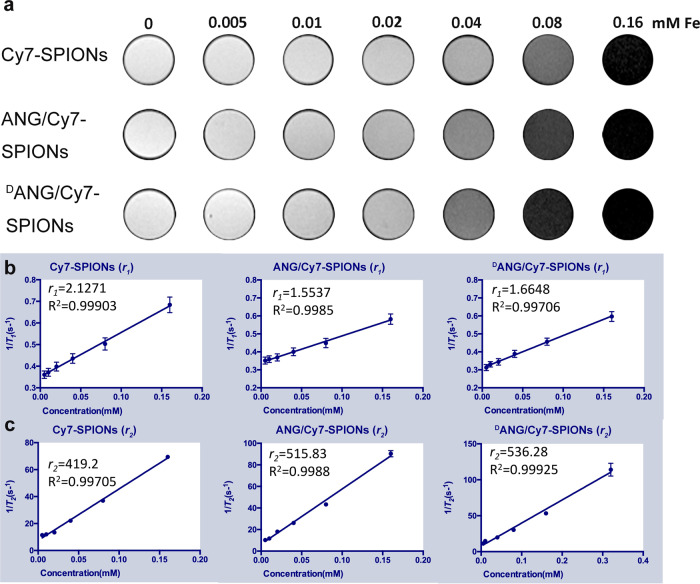
**a***T*_2_-weighted MRI of different concentrations of Cy7-SPIONs and peptide/Cy7-SPIONs probes in a 7.0 T magnetic field; **b** The *r*_1_ value of the Cy7-SPIONs probe and two peptide/Cy7-SPIONs probes in a 7.0 T magnetic field; **c** The *r*_2_ value of the Cy7-SPIONs probe and two peptide/Cy7-SPIONs probes in a 7.0 T magnetic field

For *T*_2_-weighted MR relaxometry of peptide/Cy7-SPIONs, the transverse relaxation (*r*_2_) value, longitudinal relaxation (*r*_1_) value and *r*_2_ to *r*_1_ ratio are effective indexes that determine the imaging efficiency of MRI contrast agents. As shown in Fig. 3a, the signals decreased as the Fe concentration in the probe solution increases. The *r*_2_ values of Cy7-SPIONs, ANG/Cy7-SPIONs, and ^D^ANG/Cy7-SPIONs were 419.2 S^−1^ mM^−1^, 515.83 S^−1^ mM^−1^, and 536.28 S^−1^ mM^−1^, respectively; the corresponding *r*_1_ values were 2.1271 S^−1^ mM^−1^, 1.5537 S^−1^ mM^−1^ and 1.7444 S^−1^ mM^−1^ (Fig. 3b and c). Consequently, an *r*_2_ to *r*_1_ ratio under 7.0 T was calculated for each type of probe, and the values were 197.07, 322.00, and 301.22, suggesting that all probes modified by peptides had a relatively good *T*_2_WI enhancing effect. Evidently, they were better than probes without peptide modification, possibly because the short peptides acted as hydrophilic chain segments and increased the exchange of water molecules on the surfaces of the nanoprobes.

### Cytotoxicity and in vivo toxicology

In vitro cytotoxicity was assessed by the proliferation rate of HUVECs and U87-MG cells using CCK-8 kits. These two types of cells incubated with two types of peptide/Cy7-SPIONs probes at concentrations ranging from 0 to 50 *µ*g/mL for 24 h showed a negligible proliferation change (supplementary Fig. S4a and b).

Histological examination was conducted to evaluate the in vivo long-term toxicity of the SPIONs probes. Compared with those of the control group, H&E-stained slices of the main organs (heart, liver, spleen, lung, and kidney) from healthy BALB/c mice injected with the peptide/Cy7-SPIONs suspension showed no noticeable abnormalities or tissue damage 1, 3 and 7 days after the mice were sacrificed, which indicated that the designed probes displayed nonvisible effects in vivo (supplementary Figs. S5–S7).

### Cellular affinity and in vivo biodistribution of peptides

**Fig. 4:**
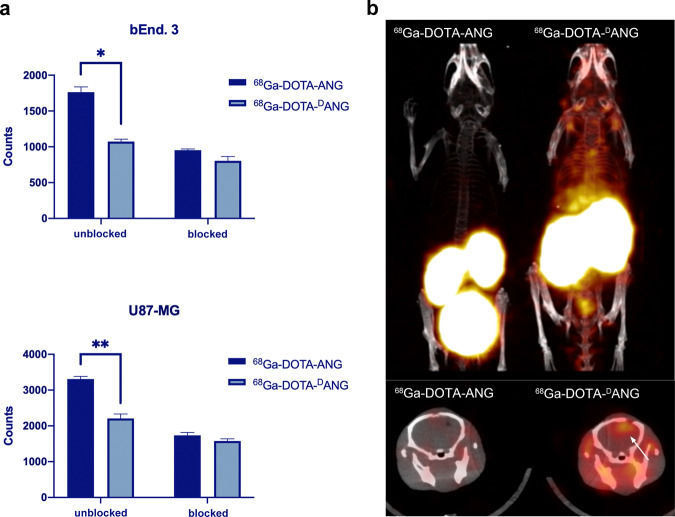
**a** Radioactivity counts of bEnd.3 and U87-MG cell lysates; **b** PET/CT images of tumour-bearing mice The upper image is a scintigraph of the in vivo distribution of probes, and the lower image is a fused presentation to show the aggregation of probes in the brain. Data from at least three independent experiments are shown as the means ± SEMs, *n* = 3, *p, **p < 0.05

The in vitro radioactive cellular uptake of ^68^Ga-DOTA-ANG or ^68^Ga-DOTA-^D^ANG peptides in glioma cells was investigated to evaluate the affinity of ANG and ^D^ANG for the LRP-1 receptor on the cell membrane. ^68^Ga-DOTA-ANG achieved better accumulation than ^68^Ga-DOTA-^D^ANG in both bEnd.3 and U87-MG cells after 2 h of incubation. Preincubation of cells with a 20-fold excess of nonlabelled peptides dramatically decreased the radioactive signal from the radiolabelled conjugates, suggesting that the LRP-1 receptor was blocked and that the uptake of ANG peptides was specifically based on these receptors (Fig. 4a).

Although ^D^ANG showed less affinity than ANG for cellular uptake, a head-to-head micro PET/CT imaging comparison indicated that ^D^ANG would be better for in vivo use (Fig. 4b). ^68^Ga-DOTA-^D^ANG and ^68^Ga-DOTA-ANG were injected separately into mice with in situ xenografted U87-MG tumours, and PET/CT images were acquired 1 h post injection. The renal bladder should be confirmed as the major metabolic route for these two kinds of peptides. ^68^Ga-DOTA-ANG exhibited faster clearance from the body, in <15 min, and did no ideal visible time window during this period was observed. On the other hand, the unnatural optical activity of ^D^ANG significantly improved the stability and biodistribution of the tracers to acquire high-quality images of tumours in the brain.

### In vitro BBB model

**Fig. 5:**
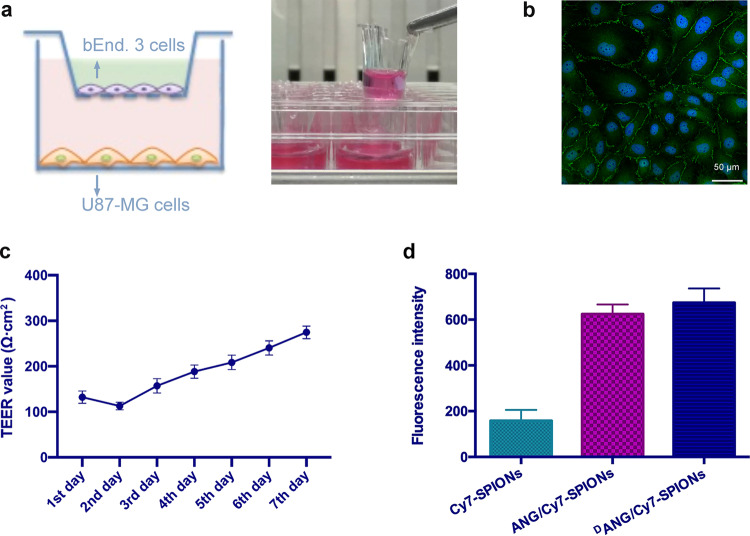
**a** Schematic of the in vitro BBB model by Transwell assays; **b** Immunofluorescence staining of the endothelial cell interstitial protein ZO-1 (green); nuclei (blue); **c** TEER value of the in vitro BBB model for 7 consecutive days; **d** The fluorescence intensities of the Cy7-SPIONs, ANG/Cy7-SPIONs, and ^D^ANG/Cy7-SPIONs probe solutions passing through the upper chamber of the Transwell unit. Data from at least three independent experiments are shown as the means ± SEMs, *n* = 3

The permeability of the model was assessed by several tests. In the leak test, the liquid levels in the upper chamber and the lower chamber did not have noticeable changes for several hours, which possibly indicated that the connection between the endothelial cells was very tight. In addition, the tight connection between endothelial cells was confirmed with immunofluorescence analysis (Fig. 5b).

For transendothelial electrical resistance (TEER) measurement, the final in vitro BBB model TEER value was recorded in the following 10 days. As shown in Fig. 5c, the value reached ~300 Ω cm^2^, indicating that this model can effectively simulate some characteristics of the blood–brain barrier of rodents.

Another test with NaFl showed that the NaFl Papp of the BBB model group was (11.24 ± 0.73) × 10^−6^ cm/s, which was significantly different from that of the blank group (Papp = 67.80 × 10^−6^ cm/s). These results indicated that the in vitro BBB model had a strong ability to limit permeability.

### Penetrability of probes in vitro

The ability of probes to penetrate the in vitro BBB model was evaluated by measuring the fluorescence intensity of probes modified by peptides in the solution passing through the upper chamber in the Transwell and comparing the fluorescence intensity of probes without peptide modifications in the control group. 60 min after adding the same amount of the probes to the upper chamber, the fluorescence intensities of the Cy7-SPIONs, ANG/Cy7-SPIONs, and ^D^ANG/Cy7-SPIONs probe solutions passing through the upper chamber were 158 ± 47.53, 624.6 ± 41.6, and 674 ± 62.22, respectively (Fig. 5d). Apparently, there was a significant difference (*p* < 0.05) between the peptide-modified groups and the control group, which indicated that the penetrating ability of the probes was significantly improved after peptide modification.

### Cellular internalization analysis

**Fig. 6:**
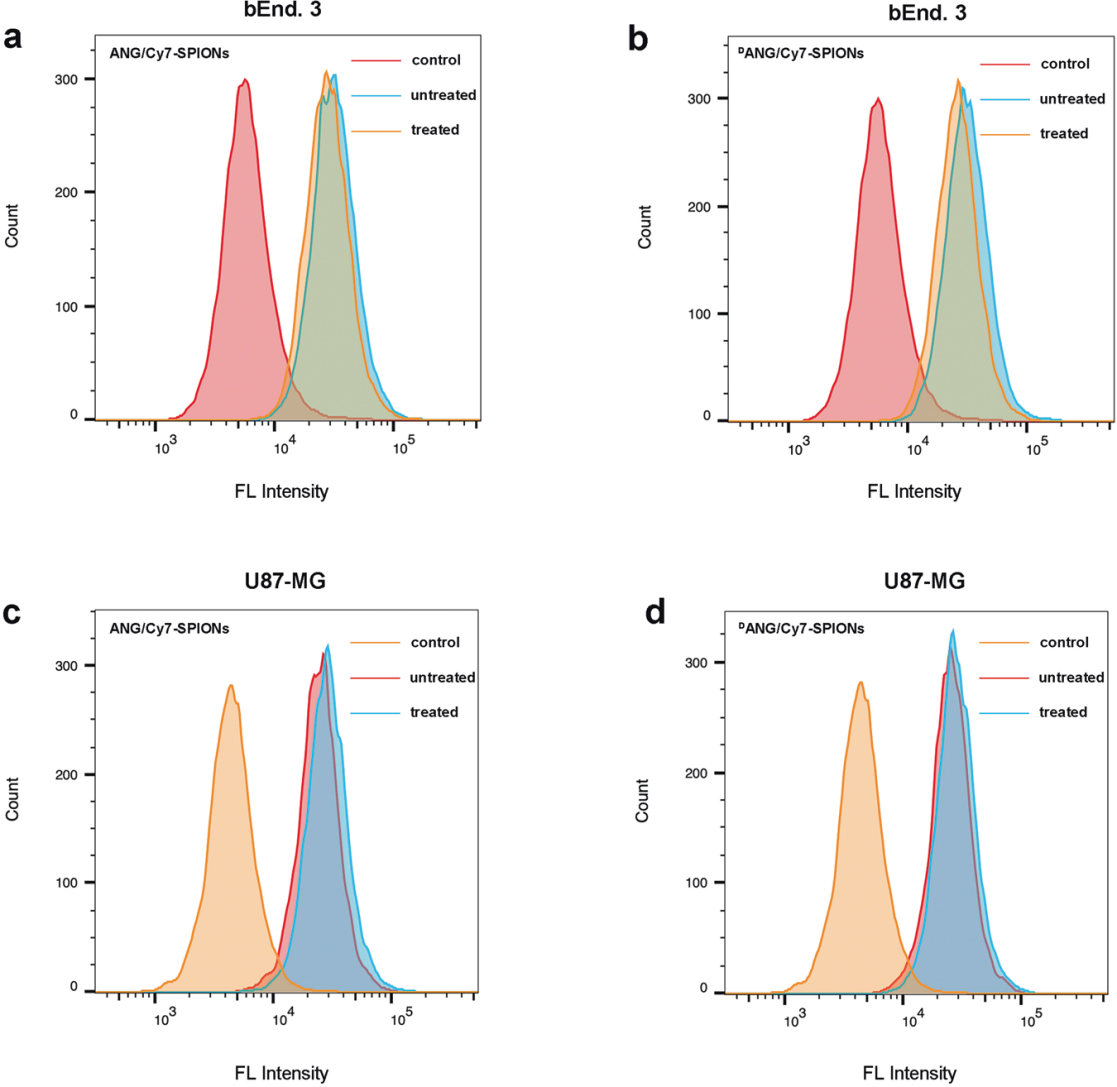
**a** FCM profiles displaying cellular uptake of the ANG/Cy7-PEG-SPIONs probes before and after incubation with human serum after culturing for 4 h with bEnd.3 cells; **b** FCM profiles displaying cellular uptake of the ^D^ANG/Cy7-PEG-SPIONs probes before and after incubation with human serum after culturing for 4 h with bEnd.3 cells; **c** FCM profiles displaying cellular uptake of the ANG/Cy7-PEG-SPIONs probes before and after incubation with human serum after culturing for 4 h with U87-MG cells; **d** FCM profiles displaying cellular uptake of the ^D^ANG/Cy7-PEG-SPIONs probes before and after incubation with human serum after culturing for 4 h with U87-MG cells. Data from at least three independent experiments are shown as the means ± SEMs, *n* = 3

We conducted quantitative analysis using flow cytometry (FCM). As shown in Fig. 6 and Supplementary Fig. S8, all peptides/Cy7-SPIONs probes could be effectively internalized by U87-MG cells and bEnd.3 cells, independent of preincubation, and the cellular uptake increased as the cells and probes were cocultured for increased times. In addition, in both U87-MG cells and bEnd.3 cells, there was no significant difference between the fluorescence intensity of preincubated probes and probes without preincubation, indicating that the structure of all probes remained stable under serum-containing conditions, which is beneficial for further in vivo experiments.

### In vivo MRI of intracranial gliomas

**Fig. 7:**
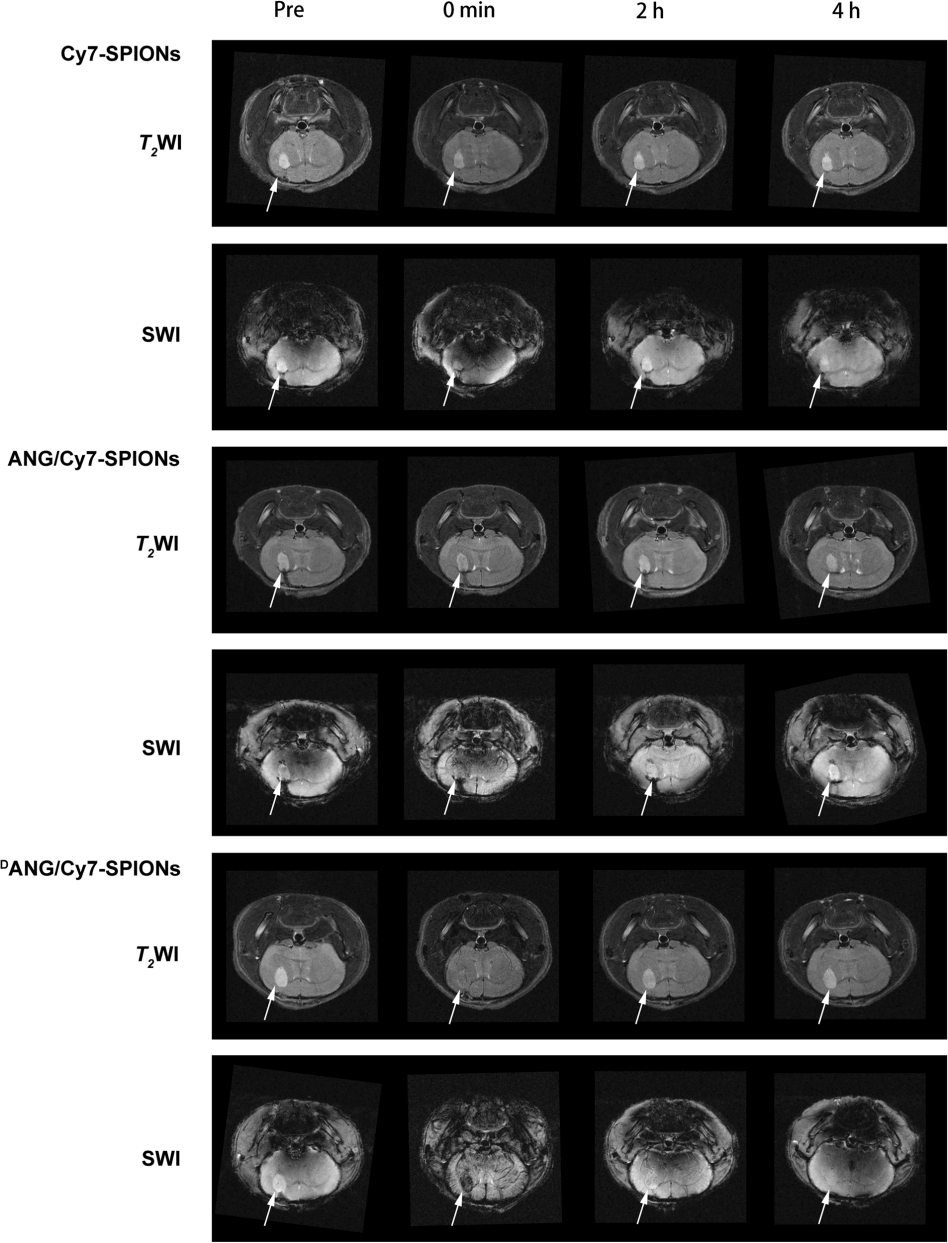
MRI of Cy7-SPIONs, ANG/Cy7-SPIONs and ^D^ANG/Cy7-SPIONs probes in tumour-bearing nude mice at different times. The upper images are *T*_2_WI, and the lower images are SWI. Gliomas are indicated by white arrows

After the tumour-bearing BALB/c nude mice were divided into several groups injected with different probes, they underwent both *T*_2_WI and SWI (Fig. 7). The low signal in gliomas weakened gradually in both *T*_2_WI and SWI as time went by, and the concentration of SPIONs inside gliomas was more distinguishable in SWI, which helped confirm the boundary of the gliomas.

As expected, different probes performed divergently in this section. For the control group injected with Cy7-SPIONs probes, effective enhanced MRI images were not acquired. The contrast-enhanced effects of gliomas in *T*_2_WI and SWI images were obvious immediately after the injection of ANG/Cy7-SPIONs and ^D^ANG/Cy7-SPIONs probes. Notably, the probes could cause the signals of gliomas and normal brain tissue to be displayed divergently in SWI images. Especially with the ^D^ANG/Cy7-SPIONs probe, the contrast-enhanced effects clearly distinguished the gliomas from the normal brain tissue via SWI, and the edges of the gliomas were also clearly visible. Then, the contrast-enhanced effects decreased rapidly in both groups injected with ANG/Cy7-SPIONs and ^D^ANG/Cy7-SPIONs probes, and disappeared after 4 h.

### Intraoperative navigation

**Fig. 8:**
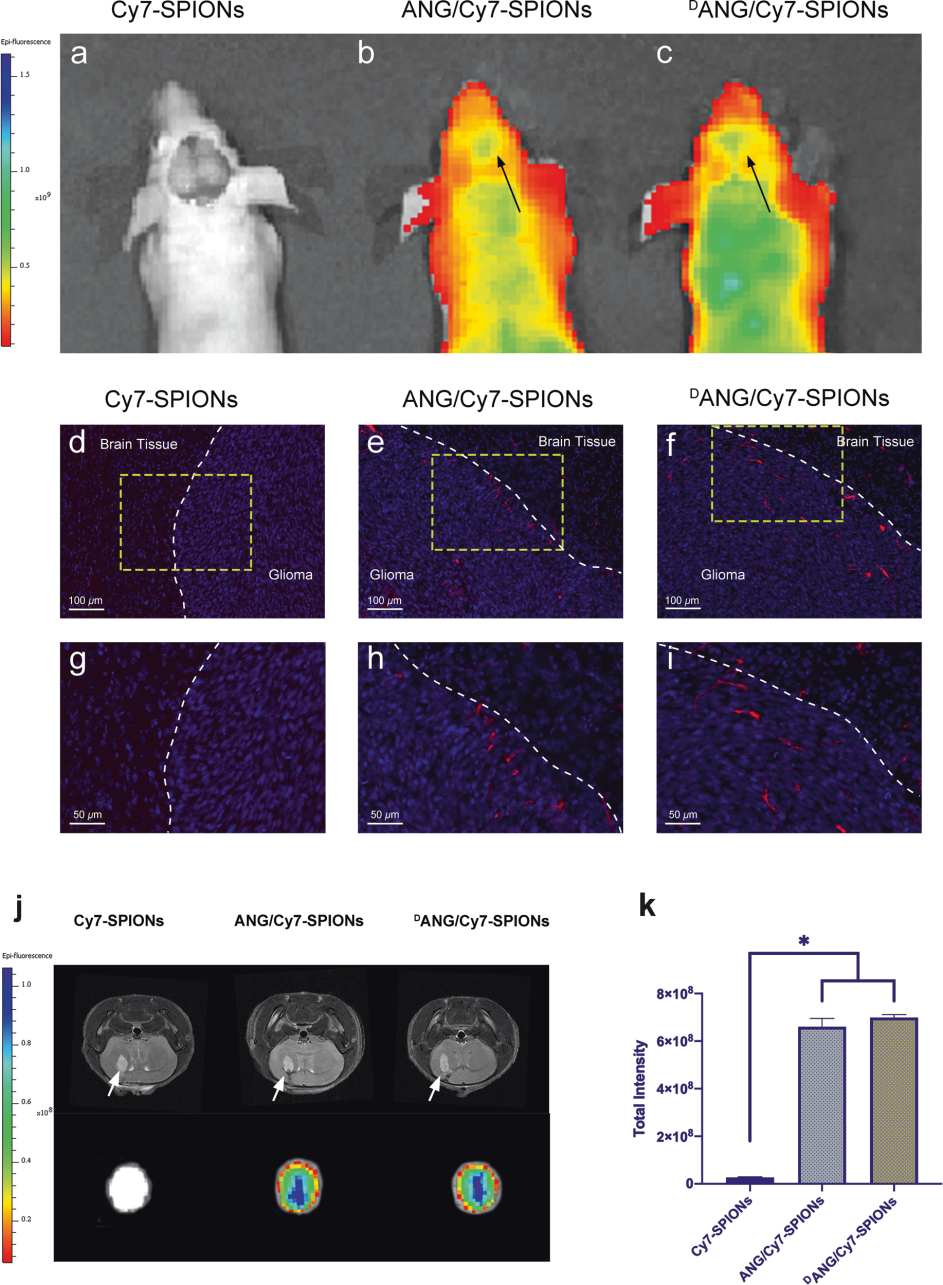
**a** In vivo images of tumour-bearing mice injected with Cy7-SPIONs, **b** ANG/Cy7-SPIONs, and **c** ^D^ANG/Cy7-SPIONs probes which were conducted with an IVIS imaging system. Images of glioma-containing brain slices from mice injected with **d** Cy7-SPIONs, **e** ANG/Cy7-SPIONs, and **f** ^D^ANG/Cy7-SPIONs which was conducted with an automated quantitative pathology imaging system. **g**–**i** The images in the lower showed a ×2 magnification of the areas in the yellow boxes in the images above. The border of the glioma is highlighted by the white dotted line. The DAPI signal in blue indicates cells, and the Cy7 signal in red indicates probes. **j** Ex vivo NIR fluorescence images of three probes in tumour-bearing brains after intravenous injection for 24 h. The upper images are MRI images showing tumour size; the lower images are NIR fluorescence images showing the fluorescence signal of the probes; **k** The total intensity of the three probes in tumour-bearing brains. Data from at least three independent experiments are shown as the means ± SEMs, *n* = 3, *p < 0.05

For in vivo intraoperative imaging of gliomas, compared with mice injected with Cy7-SPIONs probes, those injected with ANG/Cy7-SPIONs or ^D^ANG/Cy7-SPIONs probes showed completely different imaging characteristics (Fig. 8a–c). In detail, as pointed out by the arrows, the gliomas were highlighted on the images of the latter ones (Figs. 8b and c), which meant that the peptides could help the probes stay longer in the body and target the glioma area. To further illustrate that the probes modified with peptides could accurately show the edge of the tumour under intraoperative conditions, fluorescence imaging of tissue sections via an automated quantitative pathology imaging system was also performed. The results were consistent with the previous experiment. Peptide-modified probes, including ANG/Cy7-SPIONs and ^D^ANG/Cy7-SPIONs probes (denoted by red dots in Fig. 8d–i), could accumulate at the border of the gliomas, while the non-tumour area had almost no fluorescence signal from the probes. This difference was quite significant. On the other hand, the signals from Cy7-SPIONs probes were thoroughly undetectable (Figs. 8d and g).

### Ex vivo NIR fluorescence imaging of intracranial gliomas

Ex vivo NIR fluorescence imaging was another method to confirm whether the probes we designed had successfully targeted the intracranial gliomas. The tumour size of tumour-bearing mice was confirmed by 7.0 T MRI. Then, NIR fluorescence imaging detected the aggregation of probes at glioma sites, and then the total fluorescence intensity at each site was calculated, which was effective for evaluating the average aggregation of probes in gliomas. Cy7-SPIONs probes had the lowest aggregation at glioma sites, while both ANG/Cy7-SPIONs and ^D^ANG/Cy7-SPIONs probes had clear aggregation at glioma sites, indicating that peptide modification was effective (Fig. 8j and k). The aforementioned differences were significant (*p* < 0.05).

### Distribution of the probes in the main organs

The distribution in the brain was confirmed by scrutinizing slices of fixed brain tissue stained with Prussian blue, and the results showed that all probes aggregated at the margin of the tumours and that the probes modified by peptides had obviously higher aggregation than the unmodified probes (supplementary Fig. S9), which is consistent with the MRI and intraoperative navigation results.

The distribution in the other organs was confirmed by both fluorescence analysis and ICP-MS. As shown in Supplementary Fig. S10a, fluorescence analysis of mice sacrificed 1 h after injection showed that the probes aggregated mainly in the liver and kidney. Furthermore, ICP-MS, based on counting the iron content in the organs, was performed on healthy mice without glioma that was sacrificed 24 h after injection. The results demonstrated that there were obvious aggregations of iron in the liver and kidneys, and the spleen had the highest specific iron content (Supplementary Fig. S10b).

## Discussion

We successfully designed and produced a novel nanoprobe by simultaneously combining a NIR dye (Cy7) and retro-enantio angiopep-2 with PEG-DSPE-SPIONs. These nanoprobes show a dual-modal imaging capability that utilizes both MRI and NIR fluorescence imaging. Compared with previous methods, the clever synthesis method for these probes enables them to possess the key functions of glioma molecular probes, such as BBB penetration ability, glioma-targeted binding and a favourable safety profile.^35^ In addition, the design of dual-modality imaging allows both precise imaging and real-time imaging capabilities, eliminates the limitations of a single imaging method, and has the potential to be developed for intraoperative navigation.

First, we used a well-designed method to synthesize the nanoprobes. In our study, we encased the SPIONs with DSPE-PEG to form PEG-DSPE-SPIONs, in which DSPE-PEG, because of its distinct hydrophilic-hydrophobic segment and self-assembly ability, could effectively mediate the formation of an iron oxide core, maintain its colloidal stability and monodispersity in an aqueous solution and prolong the in vivo circulation time of SPIONs.^36^ The introduction of Cy7 molecules helps facilitates the dual-modal imaging capability of the nanoprobes, and the peptides (ANG/^D^ANG) allow the nanoprobes to penetrate the BBB and target glioma tissue.

The ^D^ANG peptide is a retro-enantio isomer of angiopep-2 (ANG), and it can also specifically bind to LRP-1 and then deliver the nanoprobes by receptor-mediated transcytosis. Compared with ANG, ^D^ANG has a weaker receptor affinity. However, thanks to its unnatural structure, it is resistant to protease degradation, hence acquiring a longer metabolism time in vivo to prolong the functional time of the nanoprobes, as proven by the former PET/CT assay. The results above are consistent with those in a previous report.^37^ The successful construction of the in vitro BBB model in our research allowed us to test the penetrability mediated by ^D^ANG, and the positive result shown in the aforementioned Transwell assay suggested excellent BBB penetrability of ^D^ANG/Cy7-SPIONs. In the cellular uptake assay, the performance of ^D^ANG/Cy7-SPIONs was similar to that of ANG/Cy7-SPIONs, while both performed significantly better than Cy7-SPIONs, indicating the ideal mediatory function of ^D^ANG for cellular internalization. All of these factors ensure the good performance of ^D^ANG-modified probes in the in vivo tests.

Application for MRI is a core requirement for our probe. To optimize the performance of our probe, we used two MRI sequences in the test, *T*_2_WI and SWI. *T*_2_WI is a basic magnetic resonance sequence and is widely used clinically.^38^ SWI uses the inhomogeneity of the magnetic field in the tissue to form an image, and is thus extremely sensitive to variations in the magnetic field.^39^ SPIONs probes exploit the characteristics of *T*_2_WI and SWI, and their aggregation in the tissue can strongly enhance the contrast of the image. In our research, ^D^ANG/Cy7-SPIONs probes were accurately targeted to gliomas and effectively enhanced their contrast to display a relatively dark signal on the image. This contrast enhancement effect was significantly better than that of either ANG/Cy7-SPIONs or Cy7-SPIONs probes, and it extended to the tumour edges, an ability that could not be achieved by the latter two probes. Consequently, ^D^ANG/Cy7-SPIONs probes can be used for precise imaging and provide guidance for precise surgical resection of glioma.

Considering the advantages of NIR fluorescence imaging dyes in intraoperative navigation and fluorescence imaging, we introduce Cy7 dye to the probe, which help ^D^ANG/Cy7-SPIONs probes acquire the capability of real-time fluorescence imaging and evolve into a dual-modal probe. In the corresponding experiments described in the previous sections, the inherent functions and superiority of the ^D^ANG/Cy7-SPIONs were shown. In vivo and ex vivo NIR fluorescence imaging successfully showed the location of the tumour, indicating that the ^D^ANG/Cy7-SPIONs probe can be used for intraoperative navigation. Furthermore, ex vivo NIR imaging of the isolated brain further reflected the function of intraoperative navigation. Consistent with the results of the MRI assay, the ^D^ANG/Cy7-SPIONs probe performed better and accumulated to higher levels in glioma margins than either the ANG/Cy7-SPIONs or Cy7-SPIONs probe, especially the latter, which showed almost no aggregation inside gliomas and exhibited an extremely fast metabolism. Specifically, ^D^ANG/Cy7-SPIONs could show the edge of the tumour when the tumour tissue is exposed, which helps to effectively remove the tumour tissue during the operation. Such a practice provides an important reference for the clinical translation of such probes in the future.

Finally, the biosafety and biodistribution of the probe also need to be considered. Gadolinium-based contrast agents (GBCA) are commonly used clinical MRI contrast agents, but recent studies have shown that their biosafety cannot be guaranteed, for this type of contrast agent is associated with renal fibrosis and the nervous system deposition.^40^ Thus, the development of new contrast agents is one of the ways to improve biosafety. Previous report about biosafety of iron oxide nanoparticle (IONP) underlined that the biological safety of IONP was significantly better than that of GBCA, especially it would not cause nephrogenic systemic fibrosis or had a long retention in vital organs.^41^ Our findings supported such arguments, the cytotoxicity test indicated that the probe had no cytotoxicity. The results of histological examination of the tissue showed that the probe would not cause damages to organs and tissues, indicating a very good biosafety. Finally, the biodistribution of the probe suggested that the metabolism and distribution of the probe followed normal rules and that the probe would not remain in the vital organs of the body for too long, thus minimizing its impact on the body.

## Conclusion

The current contrast agents used for diagnosis are generally restricted by their limited functions; thus, next-generation integrated contrast agents are in demand. Our group provides a novel approach to overcome this dilemma. The designed glioma-targeting ^D^ANG/Cy7-SPIONs nanoprobes possess good MRI and NIR fluorescence imaging efficiency simultaneously, which is valuable for preoperative diagnosis and intraoperatively locating gliomas. Consequently, we believe that they have the potential to stand out among next-generation glioma-targeting contrast agents.

## Material and methods

### Cell preparation and mouse models

U87-MG (human GBM cells), HUVECs and bEnd.3 (mouse brain endothelial) cells were obtained from the Institute of Basic Medical Science Chinese Academy of Medical Sciences (Shanghai, China). All types of cells were cultured in high-glucose DMEM (HyClone) containing 1% penicillin/streptomycin (HyClone) at 37 °C with 5% CO_2_.

Transfection and stable cell line generation: The luciferase plasmid provided by the State Key Laboratory of Biotherapy (Chengdu, China) was transfected into U87-MG cells using lentiviral vectors. Then, the transfected cells were selected and cultured using puromycin (2 *µ*g/mL). With limited dilution, the individual clones of the transfected cells were screened for detecting the luciferase activity by an IVIS spectrum (Caliper Life Sciences, IVIS Spectrum) for 4 weeks.

Adult male BALB/c mice that were 5–7 weeks old were purchased from the Institute of Experimental Animals, Sichuan Academy of Medical Sciences (Chengdu, China) and kept under SPF conditions. All animal experiments were performed with the approval of the Animal Care and Use Committee of Sichuan University.

### Materials

Peptides: The ANG peptides (Ac-TFFYGGSRGKRNNFKTEEY-OH) and ^D^ANG peptides (Ac-yeetkfnnrkgrsggyfft-OH) were synthesized through a standard solid-phase synthesis method, which used active ester chemistry to couple the Boc-protected amino acid with the deprotected resin. The crude products were purified by high-performance liquid chromatography (HPLC) and identified by mass spectrometry (MS).

PEG-DSPE-SPIONs: SPIONs were synthesized by using a hydrothermal method. First, 2 mmol of acetone iron, 10 mmol 1,2-diol, 6 mmol oleic acid, 6 mmol oil amide and 20 mL benzyl ether were mixed in a round-bottom flask, and the air in the reaction system was expelled by argon. Then, the mixture was heated to a constant temperature at 200 °C for 2 h and was heated to reflux at 300 °C for 1 h, and the mixture turned black. After the black mixture cooled to room temperature, ethanol was added to the system for precipitation. Then, the fluid was removed by centrifugation (10,000 × *g*, 10 min) and the precipitation was distributed to hexane. Finally, after the obtained SPION solution was centrifuged at 3000 × *g* to extract the aggregates, the DSPE-PEG 2000 AMINE molecule encased the SPIONs by hydrophobic action.

Cyanine 7 (Cy7): Cy7 was provided by Professor Ke’s team (Anaesthesiology Department, West China Hospital).

Peptide/Cy7-SPIONs: PEG-DSPE-SPIONs were pretreated in 0.02 M borate buffer at pH 8 and filtered through a 10 kDa ultrafiltration tube. Moreover, peptides (2 mg) were dissolved in 0.02 M MES buffer at pH 5.5. To activate the carboxyl group of the peptides, 1 mg EDC molecules and 0.5 mg NHS molecules were added into the solution and shaken at 180 rpm with a constant temperature at 25 °C for 25 min. After activation, the solution was centrifuged twice in 2 KDa ultrafiltration tubes to remove the unreacted EDC/NHS (3000 rpm, 5 min). Then, the activated peptide and PEG-DSPE-SPION solutions were blended together and reacted in a shaker for 2 h. When the reaction ended, the unreacted peptides were expelled by a 30 kD ultrafiltration tube. Cy7-NHS molecules (50 *µ*L, 1 mg/mL) were added to the solution, and the solution was oscillated at 4 °C in the dark overnight. At the end of the incubation, the peptide/Cy7-SPIONs probes were obtained by removing the unreacted dye molecules via an ultrafiltration tube.

^68^Ga-DOTA-peptides: DOTA-ANG and DOTA-^D^ANG were purchased from ChinaPeptides (Shanghai, China). ^68^Ga were chelated after pH adjustment (pH = 4) with sodium acetate. The reaction mixture was heated to 95 °C for 10 min, and the completeness of the reaction was checked by radio-liquid chromatography. The ^68^Ga compounds (^68^Ga-DOTA-peptides) were processed by solid-phase extraction before PET.

### Characterization of peptide/Cy7-SPIONs probes

The morphology of the SPIONs probes was visualized by transmission electron microscopy (TEM). The NH_2_-PEG-DSPE-SPIONs probe was measured by SAED. Moreover, the particle size distribution of various nanoparticles was measured by DLS (Malvern, Zetasizer 300, *n* = 3).

Hysteresis curves of the NH_2_-PEG-DSPE-SPIONs probe at 300 K were acquired by vibrating sample magnetometry (VSM, Lake Shore 7410) by using an applied field from 0 to 2 T.

The successful introduction of the peptide structure can be confirmed by comparing the decreased spectra at 280 nm before and after coupling. The spectral changes in peptide/Cy7-SPIONs and NH_2_-PEG-DSPE-SPIONs were detected by an ultraviolet spectrophotometer (UV3600, SHIMADZU). The amount of peptide remaining in the solution collected after the reaction was determined by HPLC (1260 Infinity, Agilent) and then compared with the total peptide content involved in the reaction to obtain the coupling efficiency. The introduction of fluorescent molecules was detected by a microplate reader (BioTek, Synergy Mx).

The magnetic resonance relaxation data were acquired by using a 7.0 T MRI scanner (Bruker, BioSpec 70/30, Switzerland). The probes were dissolved at gradient concentrations in PBS containing 1% agarose and were placed in a series of tubes. *T*_2*-*_weighted MRI was conducted on a 7.0 T MRI system, and the *r*_2_ value was determined by the formula 1/*T*_2_ = 1/*T*_2_^0^ + *r*_2_ C_Fe_ (1/*T*_2_^0^ means the relaxation rate of water without probes, and C_Fe_ is the mean Fe concentration).

### Cytotoxicity assessment

The cytotoxicity of the probes was analysed with a CCK-8 kit. U87-MG and human umbilical vein endothelial cells (HUVECs) were inoculated in 96-well plates with 1 × 10^4^ cells in each well and cultured at 37 °C with 5% CO_2_. After 24 h, probe-based medium with gradient Fe concentrations of 0.000, 3.125, 6.250, 12.500, 25.000 and 50.000 *µ*g/mL was added to the plate, and the cells were cultured for another 24 h. Then, the cells were washed with PBS and incubated in 100 *µ*L of CCK-8 solution (10 *µ*L of stock solution and 90 *µ*L of DMEM) for another 2 h. Finally, the absorbance was measured at a wavelength of 450 nm.

### Affinity and stability essay of peptides

For in vitro evaluation of the affinity of peptides for cells, ANG and ^D^ANG were labelled separately with ^68^Ga-DOTA. First, 5 × 10^5^ cells were plated in six-well plates overnight prior to use. Then, the cells were incubated with 74 kBq of ^68^Ga-DOTA-^D^ANG or ^68^Ga-DOTA-ANG for 1 h. In the competitive group, a 20-fold molar concentration of nonlabelled peptide was added 1 h prior to adding ^68^Ga- labelled peptide. The medium was removed after incubation. Then, the cells were washed three times with PBS and lysed with 0.1 M NaOH. All cell lysates were collected, and the radioactive particles were counted with a Wizard 2470 gamma counter (Perkin Elmer, US).

For in vivo evaluation of peptide stability, PET/CT imaging experiments were performed on an IRIS small animal PET/CT imaging system (inviscan SAS, Strasbourg, France). Each animal was injected with 3.7 MBq of ^68^Ga-DOTA-^D^ANG or ^68^Ga-DOTA-ANG via the tail vein, and imaged at 1 h post injection. PET data were acquired for 10 min and reconstructed with a three-dimensional ordered-subset expectation-maximization (3D-OSEM) algorithm with a Monte Carlo-based accurate detector model. CT acquisition was performed with 50 kV, 1 mA X-ray output and a total acquisition time of 140 s.

### Cell internalization study of probes

Preincubation of probes: To investigate the stability of probes in vitro, ANG/Cy7-SPIONs and ^D^ANG/Cy7-SPIONs probe solutions were mixed with equal volumes of human serum and preincubated in a shaker at 37 °C for 3 h.

Cellular uptake analysis: Quantitative analyses of the cellular uptake of probes were conducted by flow cytometry (FCM, BD FACS, Celesta).

The cellular uptake efficiency of probes and preincubated probes was measured by FCM. U87-MG cells and bEnd.3 cells were cultured in high-glucose DMEM containing 10% FBS and 1% penicillin/streptomycin at 37 °C with 5% CO_2_. When the cells grew in good condition, they were distributed to a 12-well plate at a density of 5 × 10^5^ cells per well. After cell adhesion, two types of 50 *µ*g Fe_3_O_4_/mL peptide/Cy7-SPIONs solutions and two types of 50 *µ*g Fe_3_O_4_/mL preincubated peptide/Cy7-SPIONs solutions were added to the medium to coculture with the cells for another 4 h, respectively. Afterward, the cells were washed with PBS and collected in 200 *µ*L PBS for FCM assays in which the signal channel was switched to APC-A750, and the acquired results were analysed by FlowJo.

### In vitro BBB penetration assays

To evaluate the penetrating ability of the probes, we constructed an in vitro BBB model and tested permeability with a method modified from a previous literature.^42^

Construction of the in vitro BBB model: U87-MG cells and bEnd.3 cells were initially cultured in high-glucose DMEM containing 10% FBS and 1% penicillin/streptomycin at 37 °C with 5% CO_2_. Moreover, a layer of a collagen solution was smeared on the inner side of the polycarbonate Transwell (3 *µ*m mean pore size, 0.33 cm^2^ surface area, Millipore, USA) and dried naturally on a clean bench for 30 min. Then, bEnd.3 cells were inoculated into the upper chamber of 24-well transwells units at a density of 5 × 10^4^ cells per well. Two days later, U87-MG cells were inoculated into the lower chamber at a density of 1 × 10^5^ cells per well and cocultured with bEnd.3 cells for one week.

In vitro BBB permeability test: The first test was the leak test, 200 *µ*L medium and 900 *µ*L medium were added to the upper chamber and the lower chamber respectively, then the fluid level in each chamber was recorded. After 4 h, the fluid level was checked to determine whether there were some changes.

TEER measurements were implemented by using a Millicell^®^ ERS-2 Electrical Resistance System (Millipore, Billerica, MA). After U87-MG cells were inoculated into the lower chamber, TEER measurements were performed once a day at the same timepoint of cell seeding for 7 consecutive days. In addition, the resistance of the blank culture transwell unit was recorded once daily. Then, the final in vitro BBB model TEER value in Ω·cm^2^ was attained by subtracting the TEER value of the blank culture transwell unit from the TEER value of the in vitro BBB model transwell unit. Results were evaluated as referenced in a previous review.^43^

In addition, immunofluorescence was used to test in vitro BBB permeability. Endothelial cells (bEnd.3 cells) from the in vitro BBB model were fixed with a 4% paraformaldehyde solution for 30 min at room temperature and washed twice with PBS. After fixation, the cells were incubated with 3% BSA in PBS for 1 h to block the nonspecific binding of antibodies. Then, bEnd.3 cells were incubated with rabbit polyclonal antibodies against ZO-1 (ZMD.437, Invitrogen) at 37 °C for 1 h. After being washed with PBS three times and incubated with FITC 488-conjugated anti-rabbit IgG (Invitrogen) at 37 °C for 1 h, the stained cells were re-washed with PBS three times. Subsequently, the cells were further stained with DAPI for 10 min and observed by confocal microscopy (Nikon, A1RMP+).

Another test taken as an example from a previous study using sodium fluorescein (NaFl) was also introduced to evaluate in vitro BBB permeability.^44^ First, NaFl was resolved in PBS to produce a series of standard solutions with NaFl concentrations of 0.05, 0.10, 0.15, 0.20, 0.25, 0.30, 0.35, 0.40, 0.45 and 0.50 *µ*g/mL. The absorbance of standard solutions was obtained by a multidetection monochrometer microplate reader, and subsequently, a NaFl concentration-absorbance curve was made. With the help of the curve, we measured the in vitro BBB permeability with NaFl. Then, 200 *µ*L NaFl solution (10 *µ*g/mL) and 900 *µ*L PBS were added to the upper chamber and the lower chamber, respectively, to continue culturing the cells at 37 °C with 5% CO_2_. A 100 *µ*L aliquot of solution from the lower chamber was extracted to a 96-well plate after 30, 60 and 90 min while maintaining 900 *µ*L of PBS in the lower chamber. Then, a multidetection monochrometer microplate reader (485/535 nm) was used to measure the amount of NaFl in the upper chamber. The apparent permeability coefficient (Papp) was calculated as follows:

Papp (cm/s) = d*Q*/d*t* × 1/(*A* × *C*_0_)

dQ/dt: the transfer rate of NaFl from the upper chamber to the lower chamber.

A: membrane surface area (0.33 cm^2^).

*C*_*0*_: initial concentration of NaFl (10 *µ*g/mL).

### Evaluation of the penetrability of the probes

After successfully constructing the in vitro BBB model, PBS was used to rinse the Transwell, 200 *µ*L of PBS with 10.0 *µ*g/mL Cy7-SPIONs (control) solution and 200 *µ*L of PBS with two types of 10.0 *µ*g/mL peptide/Cy7-SPIONs solutions were added to the upper chamber, and 900 *µ*L PBS was added to the lower chamber. After a 60-minute incubation, 100 *µ*L of solution from the lower chamber was extracted and distributed to a black 96-well plate in which a multifunctional fluorescence microplate reader (excitation = 720 nm, emission = 820 nm) was utilized to examine the fluorescence from the probes to determine how many probes had penetrated the in vitro BBB model.

### In situ glioma model

An in situ glioma model was established in BALB/c nude mice with weights of ~20 g and ages of 5–6 weeks. Luc-U87-MG cells were first cultured in high-glucose DMEM containing 10% FBS and 1% penicillin/streptomycin at 37 °C with 5% CO_2._^45^ As the cells grew well, trypsin was used to digest the cells so that the initial cell assembly broke into fragments for the following in situ glioma inoculation. Then, the mice received 5 × 10^4^Luc-U87-MG cells per mouse by injection of a 5 *µ*L single-cell suspension via surgery. Three weeks after the surgery, an IVIS imaging system was used to observe and confirm the success of the model.

### In vivo MRI

In vivo MRI studies were based on tumour-bearing nude mice in which in situ glioma models were successfully established. It was carried out on a 7.0 T magnetic resonance instrument (Bruker, BioSpec 70/30USR) by acquiring brain section images via *T*_2_-weighted imaging (*T*_2_WI) and susceptibility-weighted imaging (SWI) MRI. The parameters are listed as follows:

*T*_2_WI: TE = 33 ms; TR = 2520 ms; slice thickness (SL) = 0.6 mm; field of view (Fov) read = 20 mm; matrix size = 256 × 256; NEX: 3.

SWI: TE = 11 ms; TR = 581 ms; slice thickness (SL) = 0.6 mm; field of view (Fov) read = 20 mm; matrix size = 256 × 256; NEX: 2.

To investigate the performance of different peptide SPIONs probes in penetrating the BBB and stability in vivo, the mice were divided into 3 groups. The first group was the control group, in which Cy7-SPIONs probes (10 mg Fe/kg dose) without peptides were injected into the mice, while ANG/Cy7-SPIONs and ^D^ANG/Cy7-SPIONs probes (10 mg Fe/kg dose) were injected into the other two groups.

The mice in each group were first anaesthetized, and images of *T*_2_WI and SWI were obtained for the first time. Then, *T*_2_WI and SWI images were obtained at 0 min, 2 h, 4 h and 24 h after the injection of the probes.

### Intraoperative navigation

Tumour-bearing nude mice were divided into three groups and injected with Cy7-SPIONs, ANG/Cy7-SPIONs or ^D^ANG/Cy7-SPIONs probes. One hour later, mice were anaesthetized for craniotomy. Then, the brain tissue where the glioma was located was exposed, and the mice were imaged with an IVIS imaging system (Caliper Life Sciences, IVIS Spectrum, excitation: 720 nm; emission: 820 nm) for in vivo intraoperative imaging of the gliomas.

After finishing the in vivo imaging experiments, the isolated brains of mice were fixed for 2 days and completely dehydrated with a 30% sucrose solution three times. Then, the fixed brains were embedded with optimal cutting temperature (OCT) compound in a cryostat to obtain frozen slices. The slices were stained with DAPI and finally placed in an automated quantitative pathology imaging system (Vectra Polaris, Akoya Biosciences, USA) to complete the imaging.

### Ex vivo NIR fluorescence imaging of glioma and distribution of probes in mice

NIR fluorescence imaging of intracranial glioma required craniotomy, so the in vitro imaging method was chosen. The tumour-bearing mice were divided into three groups and received 10 mg Fe_3_O_4_/kg Cy7-SPIONs, ANG/Cy7-SPIONs or ^D^ANG/Cy7-SPIONs. One hour later, the mice were sacrificed by cardiac perfusion with saline and 4% polyformaldehyde. Then, the brains and main organs were extracted for glioma imaging, and the probe distribution in organs was observed via an IVIS imaging system (Caliper Life Sciences, IVIS Spectrum, excitation: 720 nm; emission: 820 nm).

The distribution of probes in the brain was confirmed with the following process. After finishing the imaging experiments, the isolated brains of mice were fixed for 2 days and completely dehydrated with a 30% sucrose solution three times. Then, the fixed brains were embedded with OCT compound in a cryostat to obtain frozen slices. Prussian blue was used to stain the slices for histological examination through an optical microscope.

The distribution of probes in other organs was confirmed with the following process. A number of healthy BALB/c mice without tumour implantation were divided into four groups, one of which was the control group (injected with 0.9% NaCl solution), while the other five were the experimental groups (injected with 10 mg Fe_3_O_4_/kg Cy7-SPIONs, ANG/Cy7-SPIONs or ^D^ANG/Cy7-SPIONs solutions). All mice were sacrificed 24 h after the injection, and the main organs were extracted. For the histological examination, the organs were embedded in paraffin, sectioned (4 *µ*m thick), stained with haematoxylin and eosin (H&E) and finally observed under an optical microscope. For the quantitative distribution of probes in mice, the main organs were cut up and digested in 1 mL hydrogen peroxide and 2 mL nitric acid for 2 days. The mixture was then placed in an oil bath at 120 °C until no precipitation remained. After it fully evaporated to dryness, 5 mL of 2% nitric acid was added to measure the volume. Processed by ultrasound for 20 min, 4 mL of solution was transferred to an Eppendorf (EP) tube, and the iron content was measured by inductively coupled plasma-mass spectrometry (ICP-MS, VG PQExCell, TJA, USA).

### Statistical analysis

For all the data, *p* < 0.05 was considered statistically significant. The data are presented as the means ± SEMs. ANOVA and Student’s two-tailed *t*-tests were used to analyse the data.

## Supplementary information

Supplementary Materials for Retro-enantio isomer of angiopep-2 assists nanoprobes across the blood-brain barrier for targeted magnetic resonance/fluorescence imaging of glioblastoma
