# Supplementary material for: Retro-enantio isomer of angiopep-2 assists nanoprobes across the blood-brain barrier for targeted magnetic resonance/fluorescence imaging of glioblastoma
Source: Signal Transduct Target Ther. 2021 Aug 19;6:309. doi: 10.1038/s41392-021-00724-y (PMC8377144; doi:10.1038/s41392-021-00724-y)
Supplement: Supplementary file 1 — Supplementary Materials for Retro-enantio isomer of angiopep-2 assists nanoprobes across the blood-brain barrier for targeted magnetic resonance/fluorescence imaging of glioblastoma [file 41392_2021_724_MOESM1_ESM.docx]

Supplementary Materials for

**Retro-enantio isomer of angiopep-2 assists nanoprobes across the blood-brain barrier for targeted magnetic resonance/fluorescence imaging of glioblastoma**

*Ruoxi Xie ^a, b, ‡^, Zijun Wu ^a, b, ‡^, Fanxin Zeng ^a, c, ‡^, Huawei Cai ^d^, Dan Wang ^a, b^, Lei Gu ^e^_,_ Hongyan Zhu ^e^, Su Lui ^a, b^, Gang Guo ^e^, Bin Song ^a^, Jinxing Li ^f^, Min Wu ^a, b, *^, Qiyong Gong ^a, b, *^*

1. Huaxi MR Research Center (HMRRC), Department of Radiology, Functional and Molecular Imaging Key Laboratory of Sichuan Province, West China Hospital, Sichuan University, Chengdu, 610041, China
2. Research Unit of Psychoradiology, Chinese Academy of Medical Sciences, Chengdu, 610041, China
3. Department of Clinic Medical Center, Dazhou Central Hospital, Dazhou,
   635000, China
4. Laboratory of Clinical Nuclear Medicine, Department of Nuclear Medicine, West China Hospital, Sichuan University, No. 37 Guo Xue Alley, Chengdu, 610041, China
5. State Key Laboratory of Biotherapy and Cancer Center, West China Hospital, Sichuan University, and Collaborative Innovation Center for Biotherapy, Chengdu, 610041, China

f. Department of Chemical Engineering, Stanford University, Stanford, California, 94305, USA

Correspondence to: wuminscu@scu.edu.cn

**This PDF file includes:**

Figures S1 to S10


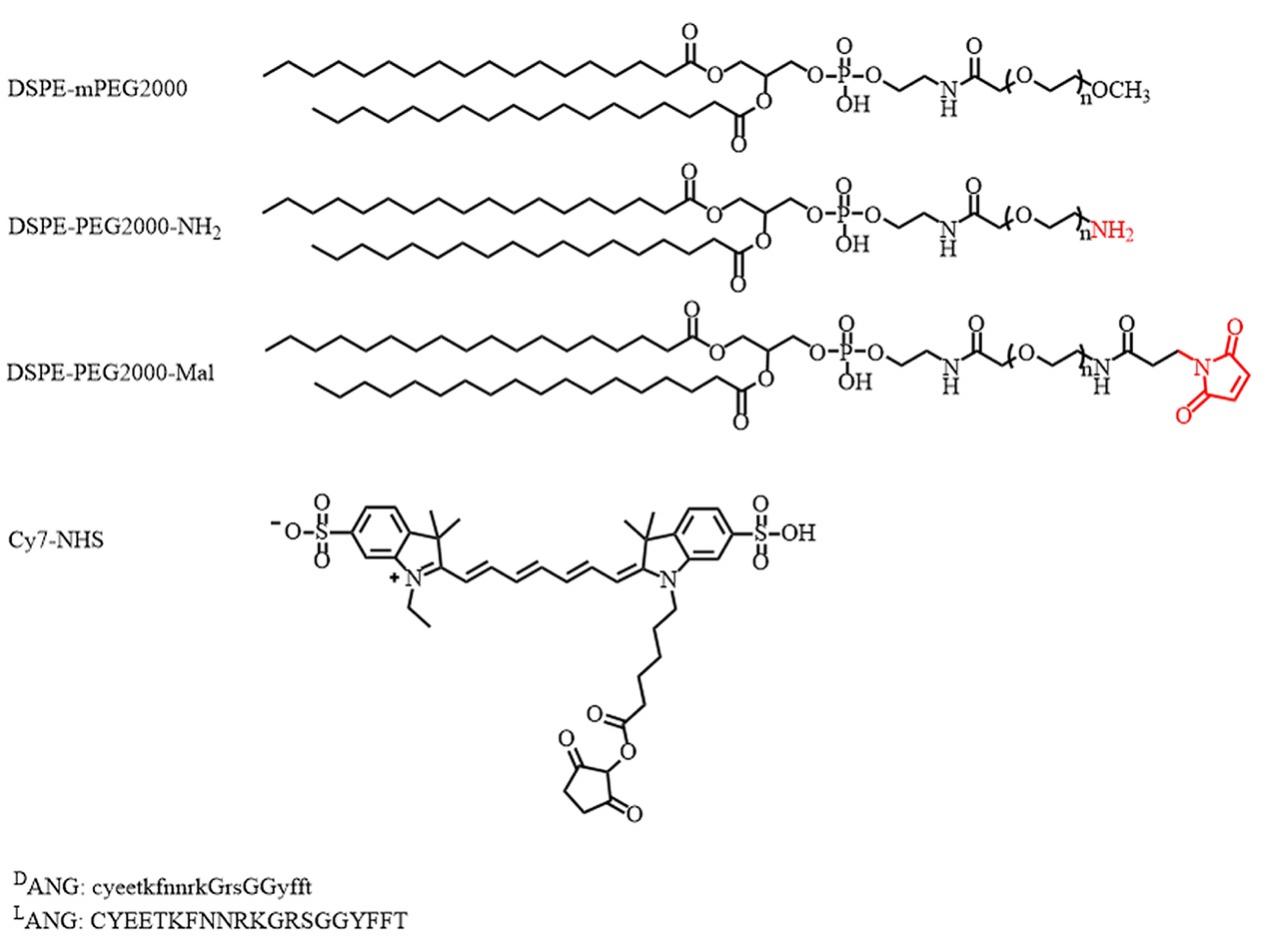


Figure S1.

Fig.S1: Chemical structures of 3 subtypes of DSPE-PEG2000, DSPE-mPEG2000, DSPE-PEG2000-NH­_2_ for Cy7 dye conjugation, DSPE-PEG2000-Mal for peptide conjugation; chemical structure of Cy7-NHS molecule; sequences of ^D^ANG and ^L^ANG.


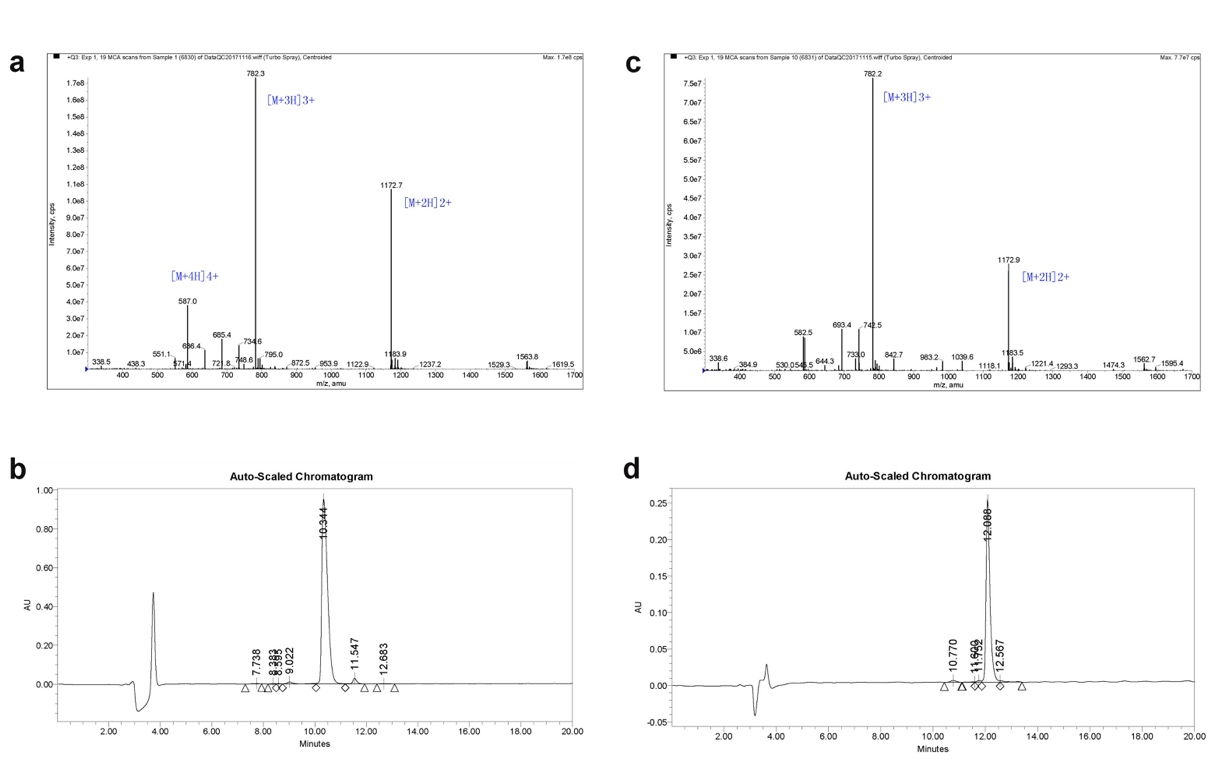


Figure S2.

Fig.S2: (a) The MS spectrum and (b) HPLC spectrum of ANG peptide; (c) the MS spectrum and (d) HPLC spectrum of ^D^ANG peptide.


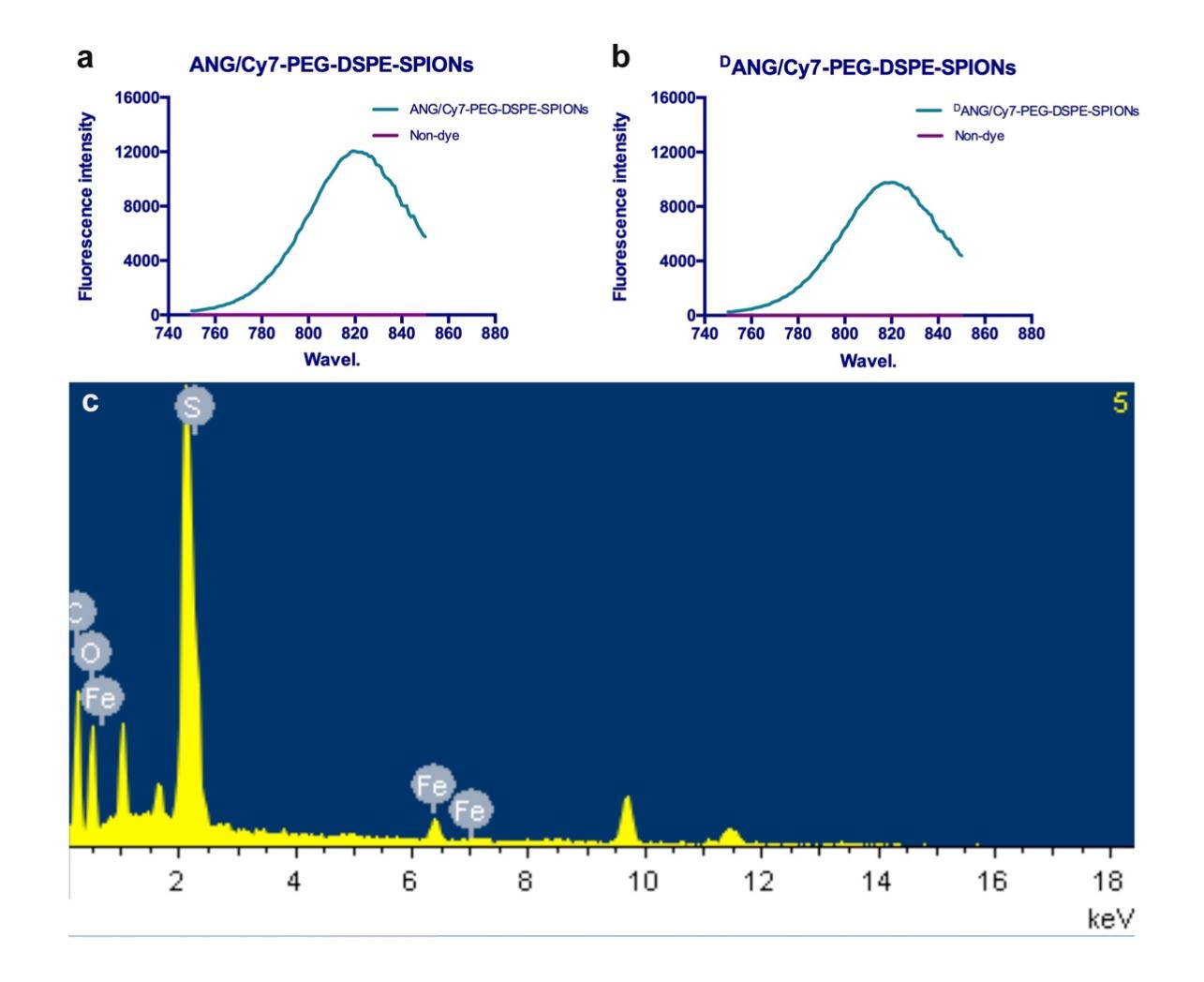


Figure S3.

Fig.S3: (a,b) The fluorescence intensity of two peptides/Cy7-SPIONs probes; (c) EDS spectrum of the nanoparticles show the related elements.


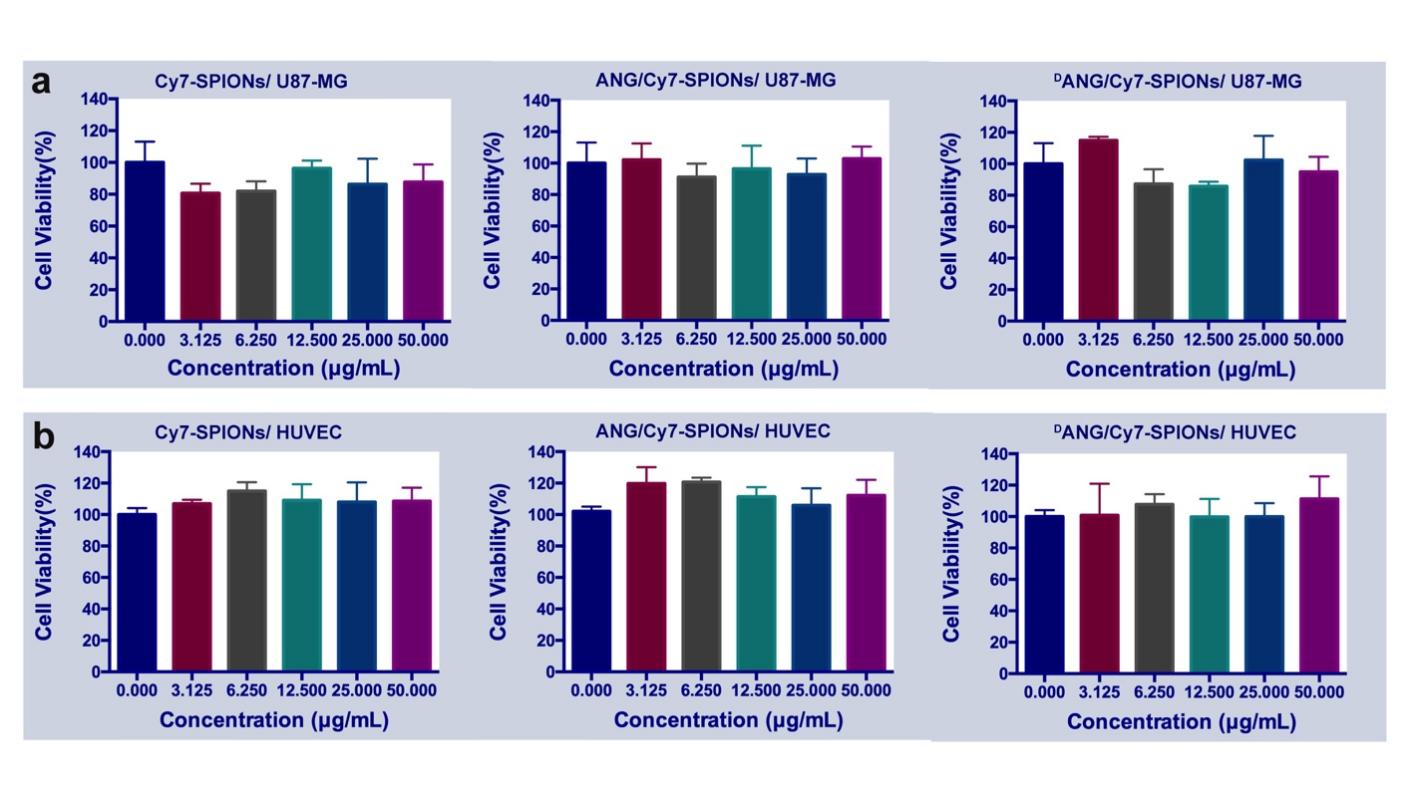


Figure S4.

Fig.S4: The cytotoxicity of Cy7-SPIONs, ANG/Cy7-SPIONs, and ^D^ANG/Cy7-SPIONs probes on U87-MG cells (a) and HUVECs (b).


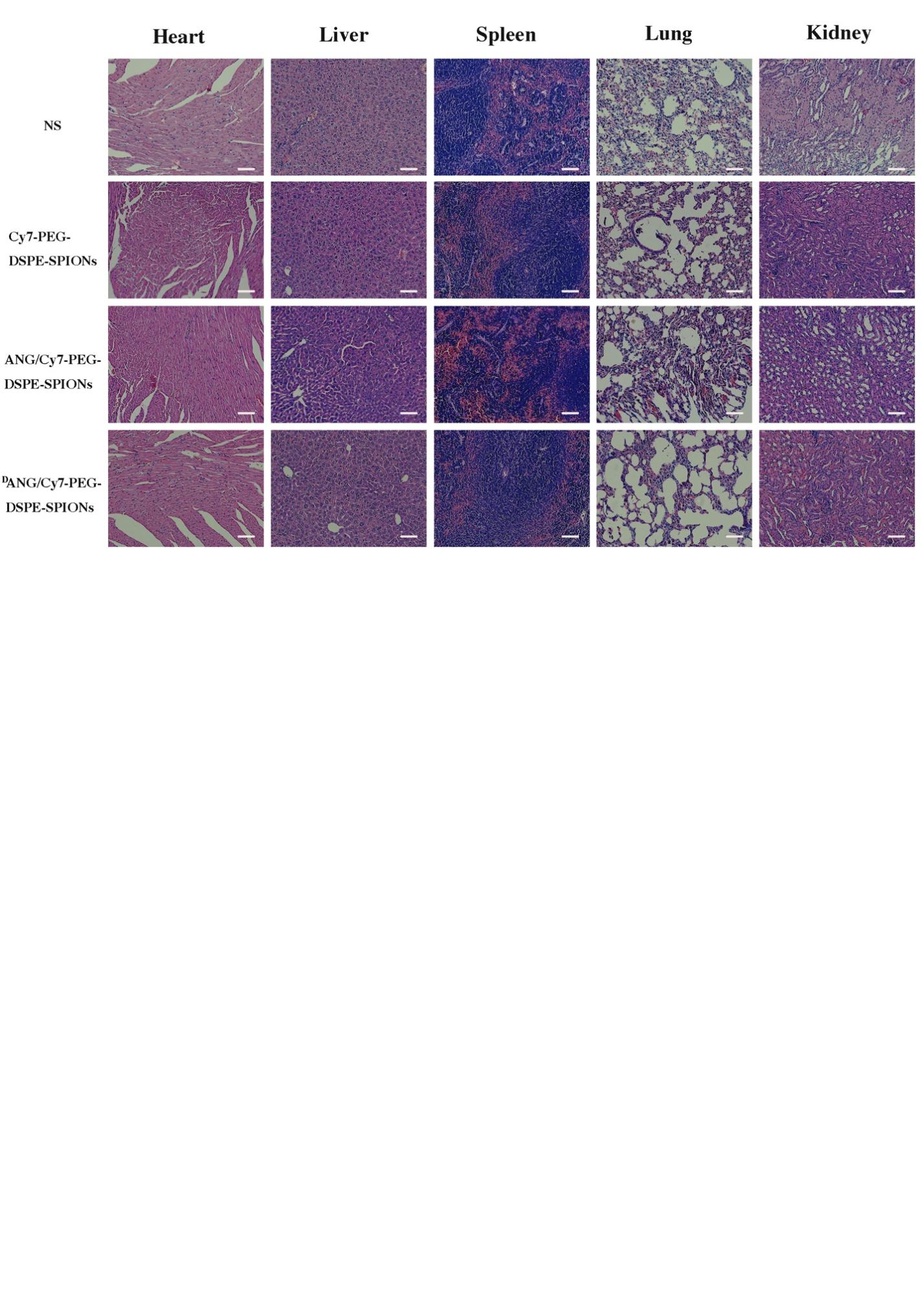


Figure S5.

Fig.S5: Histological changes of major organs at one day after a single-dose intravenous injection of Cy7-SPIONs and peptides/Cy7-SPIONs probes. (Scale bars: 50 *µ*m).


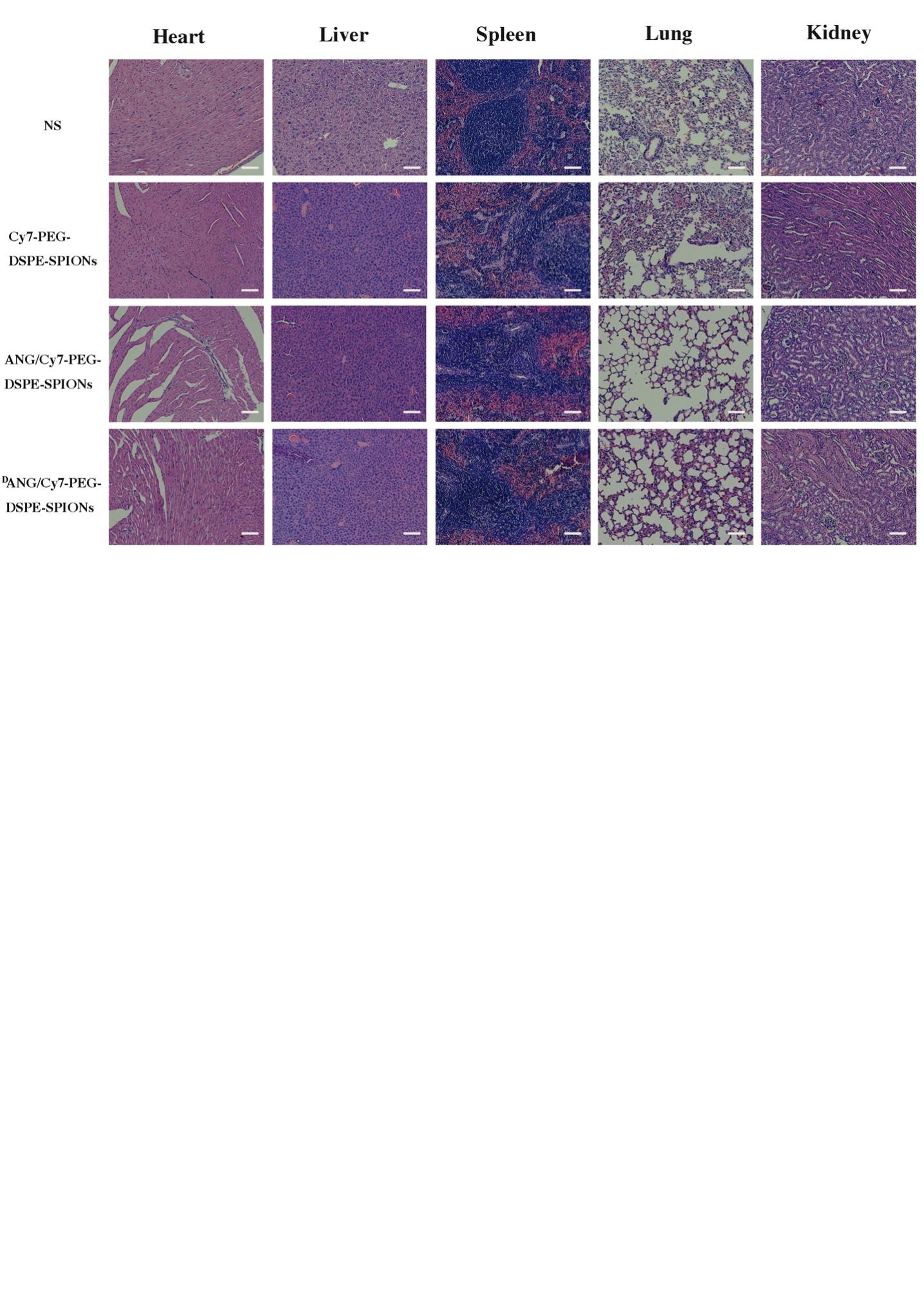


Figure S6.

Fig.S6: Histological changes of major organs at three day after a single-dose intravenous injection of Cy7-SPIONs and peptides/Cy7-SPIONs probes. (Scale bars: 50 *µ*m).


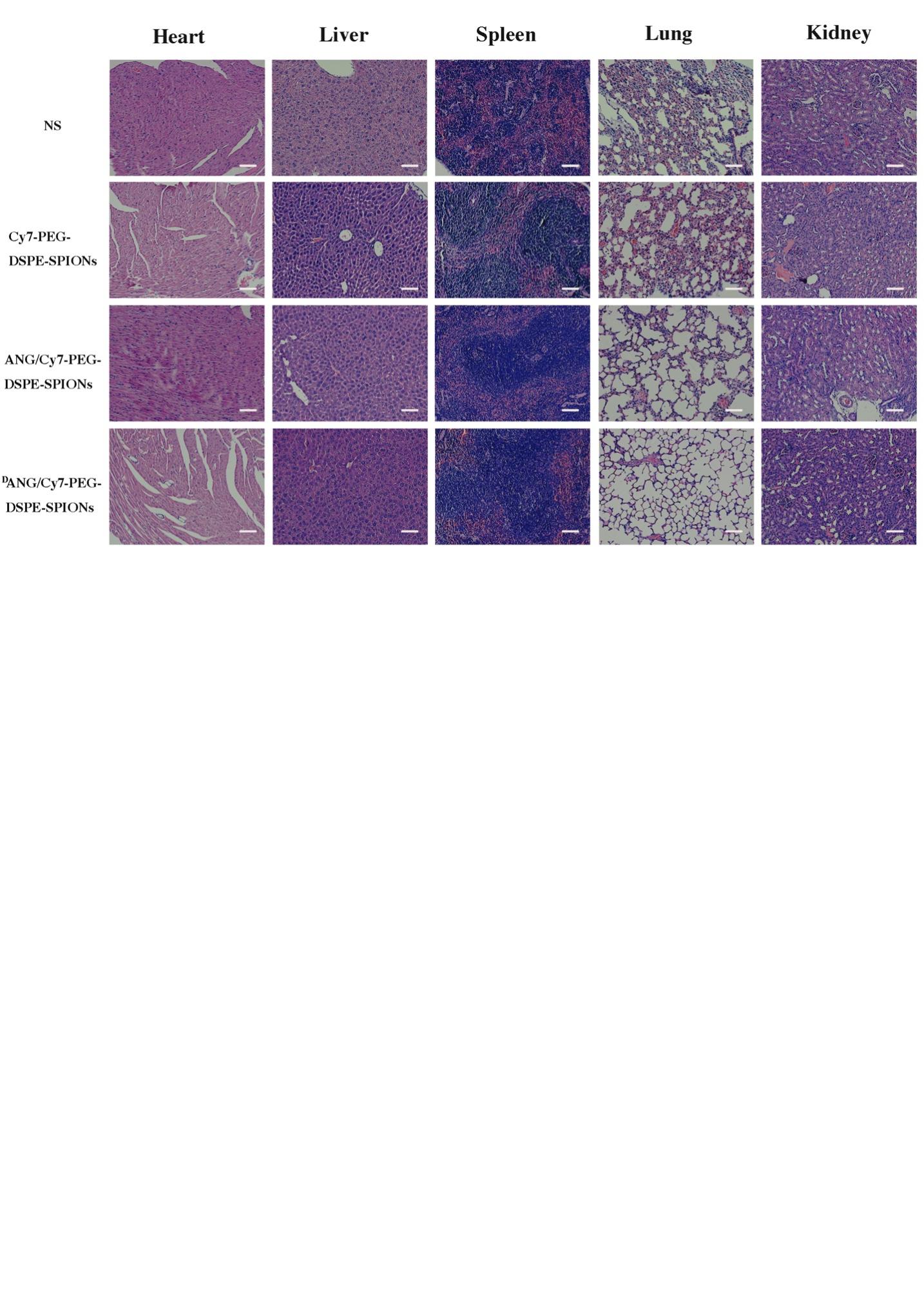


Figure S7.

Fig.S7: Histological changes of major organs at seven day after a single-dose intravenous injection of Cy7-SPIONs and peptides/Cy7- SPIONs probes. (Scale bars: 50 *µ*m).


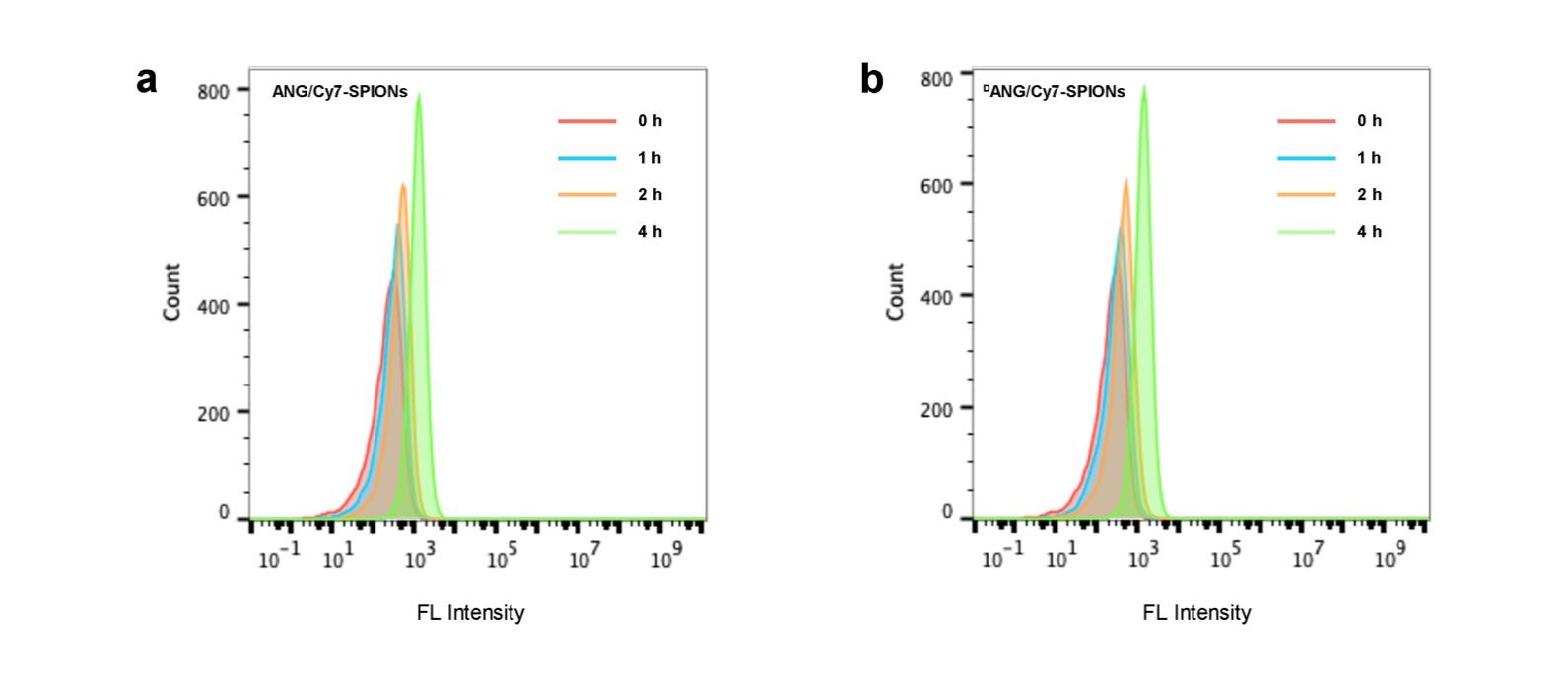


Figure S8.

Fig.S8: (a) Typical FCM profiles of cellular uptake of the ANG/Cy7-SPIONs probes by U87-MG cells; (b) typical FCM profiles of cellular uptake of the ^D^ANG/Cy7-SPIONs probes by U87-MG cells.


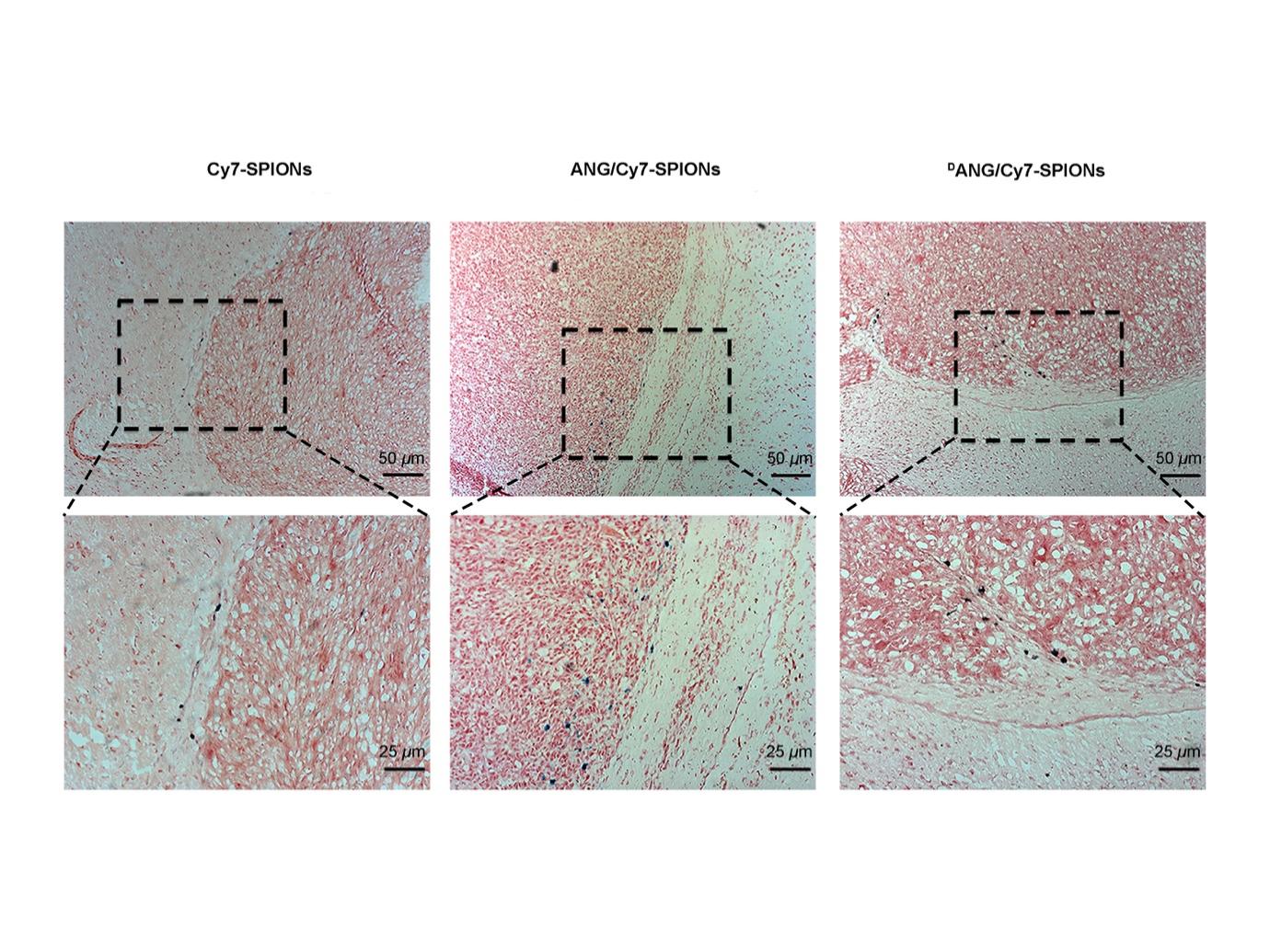


Figure S9.

Fig.S9: Prussian blue-stained brain slices show that the three types of SPIONs probes aggregate in the tumour edges after intravenous injection for 24 hours in tumour-bearing nude mice (upper scale bars: 50 *µ*m; lower scale bar: 25 *µ*m).


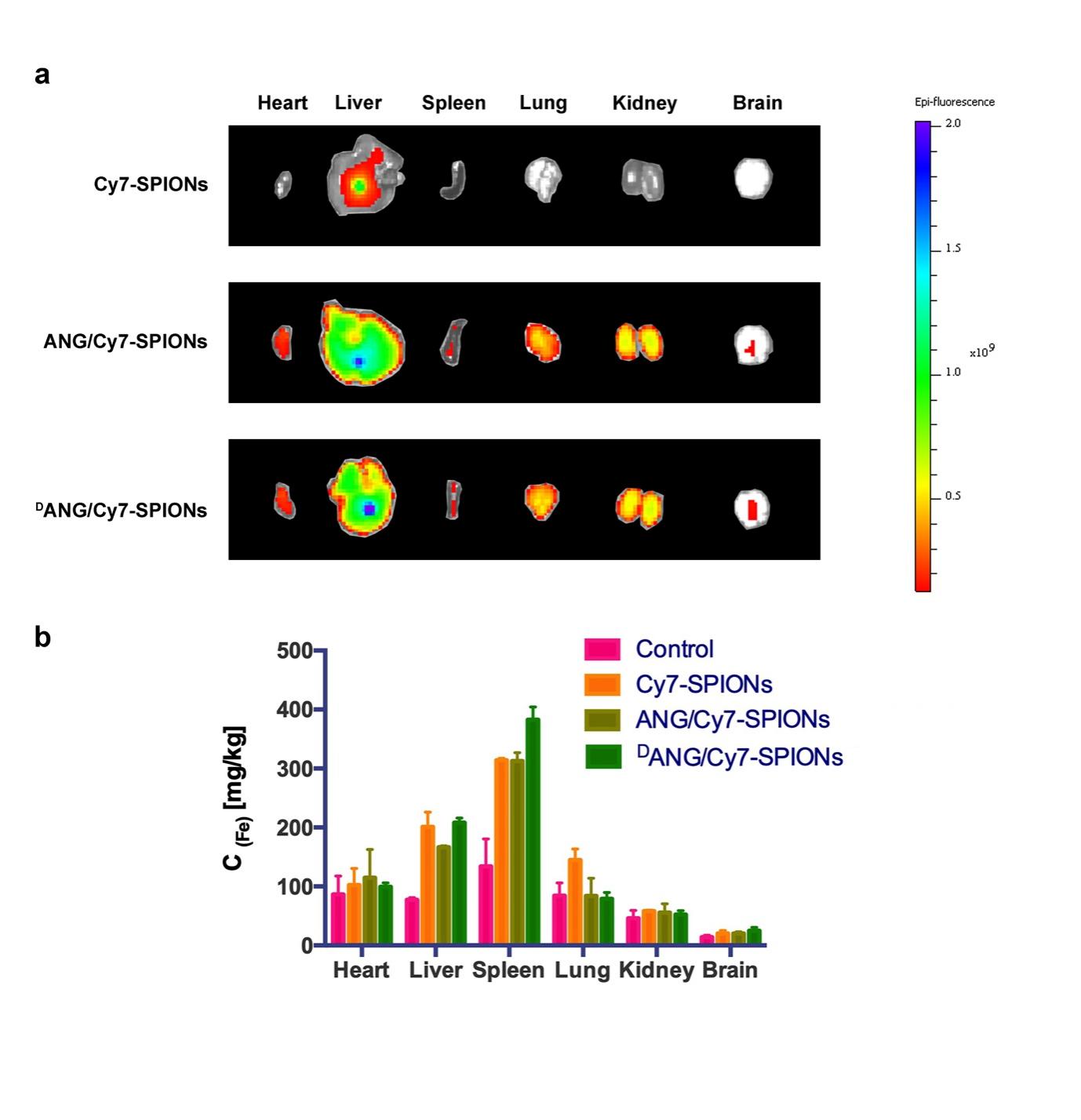


Figure S10.

Fig.S10: (a) Typical images of *ex vivo* imaging of Cy7-SPIONs probes and two peptide/Cy7-SPIONs probes on tumours and major organs resected from mice 1 hour after intravenous injection; (b) iron content analysis of different organs (heart/liver/spleen/lung/kidney/brain) from healthy nude mice injected intravenously with saline, Cy7-SPIONs probes, ANG/Cy7-SPIONs probes or ^D^ANG/Cy7-SPIONs probes after 24 hours. Data from at least three independent experiments are shown as the means ± SEMs, n = 3.
